# Hyperconnectivity of Two Separate Long-Range Cholinergic Systems Contributes to the Reorganization of the Brain Functional Connectivity during Nicotine Withdrawal in Male Mice

**DOI:** 10.1523/ENEURO.0019-23.2023

**Published:** 2023-06-26

**Authors:** Lieselot L. G. Carrette, Adam Kimbrough, Pasha A. Davoudian, Alex C. Kwan, Andres Collazo, Olivier George

**Affiliations:** 1 Department of Psychiatry, UC San Diego, California 92093; 2 Medical Scientist Training Program, Yale University School of Medicine, New Haven, Connecticut 06511; 3 Interdepartmental Neuroscience Program, Yale University School of Medicine, New Haven, Connecticut 06511; 4 Meinig School of Biomedical Engineering, Cornell University, Ithaca, New York 14853; 5 Beckman Institute, California Institute of Technology, Pasadena, California 91125

**Keywords:** addiction, Fos reactivity, single-cell whole-brain imaging, stimulant

## Abstract

Chronic nicotine results in dependence with withdrawal symptoms on discontinuation of use, through desensitization of nicotinic acetylcholine receptors and altered cholinergic neurotransmission. Nicotine withdrawal is associated with increased whole-brain functional connectivity and decreased network modularity; however, the role of cholinergic neurons in those changes is unknown. To identify the contribution of nicotinic receptors and cholinergic regions to changes in the functional network, we analyzed the contribution of the main cholinergic regions to brain-wide activation of the immediate early-gene Fos during withdrawal in male mice and correlated these changes with the expression of nicotinic receptor mRNA throughout the brain. We show that the main functional connectivity modules included the main long-range cholinergic regions, which were highly synchronized with the rest of the brain. However, despite this hyperconnectivity, they were organized into two anticorrelated networks that were separated into basal forebrain-projecting and brainstem-thalamic-projecting cholinergic regions, validating a long-standing hypothesis of the organization of the brain cholinergic systems. Moreover, baseline (without nicotine) expression of *Chrna2*, *Chrna3*, *Chrna10*, and *Chrnd* mRNA of each brain region correlated with withdrawal-induced changes in Fos expression. Finally, by mining the Allen Brain mRNA expression database, we were able to identify 1755 gene candidates and three pathways (Sox2-Oct4-Nanog, JAK-STAT, and MeCP2-GABA) that may contribute to nicotine withdrawal-induced Fos expression. These results identify the dual contribution of the basal forebrain and brainstem-thalamic cholinergic systems to whole-brain functional connectivity during withdrawal; and identify nicotinic receptors and novel cellular pathways that may be critical for the transition to nicotine dependence.

## Visual Abstract

**Figure:**
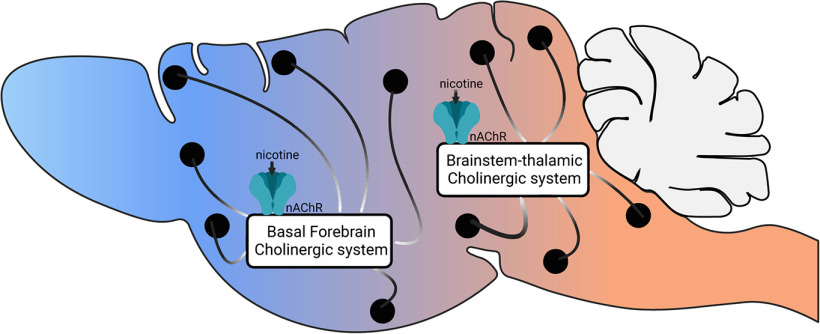

## Significance Statement

Discontinuation of nicotine use in dependent users is associated with increased whole-brain activation and functional connectivity and leads to withdrawal symptoms. Here we investigated the contribution of the nicotinic cholinergic receptors and main cholinergic projecting brain areas in the whole-brain changes associated with withdrawal. This not only allowed us to visualize and confirm the previously described duality of the cholinergic brain system using this novel methodology, but also identify nicotinic receptors together with 1751 other genes that contribute to, and could thus be targets for treatments against, nicotine withdrawal and dependence.

## Introduction

Chronic nicotine use causes adaptive changes throughout the brain that lead to drug dependence (Markou, 2008; Martin-Soelch, 2013; Fowler et al., 2020), the emergence of a withdrawal state following cessation, and long-lasting somatic and motivational symptoms (Le Foll and Goldberg, 2009) that contribute to relapse (Allen et al., 2008; Zhou et al., 2009). Brain states, like dependence and withdrawal, have been described through patterns of synchronous neural firing (Brown, 2006). Changes in the patterns of neuronal coreactivity, also called the functional connectome, can be observed in humans and rodents during withdrawal from nicotine (Hobkirk et al., 2018; Cheng et al., 2019; Kimbrough et al., 2021). Whole-brain imaging with single-cell resolution using light-sheet microscopy on cleared brains (Renier et al., 2014, 2016; Ueda et al., 2020) has made the study of brain-wide functional networks at single-cell resolution possible by looking at the expression of the immediate-early gene Fos (Wheeler et al., 2013; Vetere et al., 2017; Kimbrough et al., 2020; 2021; Smith et al., 2021; Roland et al., 2022), a marker of neuronal reactivity, which integrates neuronal activation during a period of 1–2 h, an ideal temporal window to characterize nicotine withdrawal. Using this approach, we have found that mice in withdrawal exhibit a pronounced increase in coactivation patterns throughout the brain (Kimbrough et al., 2020, 2021). A healthy control brain is modularly organized in several small, correlated clusters or modules consisting of brain regions that are functionally related. The withdrawal-induced increase in correlation between brain regions causes more regions to cluster together with a significant decrease in whole-brain modularity. Increased functional connectivity throughout the network also results in a reduction of brain regions identified as hubs. Hub regions are regions with the highest intramodular and intermodular connectivity as measured using graph theory (participation coefficient, within-module degree). These hub regions are hypothesized to be the biggest drivers of neuronal activity within the network. For instance, during nicotine withdrawal, the main hub regions shift from cortical (e.g., sensory, motor) to subcortical (e.g., amygdalar, thalamic, hypothalamic, and midbrain) regions (Kimbrough et al., 2020, 2021). However, the role of cholinergic neurons and cholinergic receptors in the whole-brain functional hyperconnectivity observed during withdrawal is unknown.

Desensitization and upregulation of nicotinic acetylcholine receptors (nAChRs) (Benwell et al., 1988; Balfour and Fagerström, 1996; Dani and Heinemann, 1996; Fowler et al., 2020) contributes to the emergence of nicotine withdrawal symptoms by altering cholinergic neurotransmission in brain regions critical to sensory processing (Gil and Metherate, 2019), attention (Hahn, 2015), emotion, and motivation (Leslie et al., 2013). nAChRs form pentameric structures assembled from a family of subunits composed of α_2_–α_10_ and β_2_–β_4_. α_4_ and β_2_ are the most prevalent, but all subunits are expressed throughout the brain. A large number of brain regions (40+) have cholinergic neurons, characterized by the expression of choline acetyltransferase (ChAT); however, most of them are interneurons, and only eight brain regions have long-range projecting cholinergic neurons (Mesulam et al., 1983). The long-range cholinergic regions include Ch1 [medial septal nucleus (MS)], Ch2 [vertical nucleus of the diagonal band (NDB)], Ch3 [horizontal limb of the NDB], Ch4 [nucleus basalis of Meynert that consists of the magnocellular nucleus (MA) and substantia innominata (SI)], Ch5 [pedunculopontine nucleus (PPN)], Ch6 [laterodorsal tegmental nucleus], Ch7 [medial habenula (MH)], and Ch8 [parabigeminal nucleus] (Mesulam et al., 1983; Woolf, 1991). We hypothesized that, following chronic nicotine administration, most cholinergic regions that are rich in nicotinic receptors would have a synchronized correlated activity due in part to the brain-wide upregulation of nicotinic receptors (Govind et al., 2009; Fowler et al., 2020) and the increase in cholinergic transmission during nicotine withdrawal (Carcoba et al., 2014). The increased correlation would lead to larger modules and decreased modularity. Furthermore, since cholinergic receptor signaling is critical for nicotine-induced Fos activation (Pang et al., 2016; Simmons et al., 2016), a subhypothesis was that the regional expression level of cholinergic-related genes would be correlated to regional differential Fos expression under withdrawal in nicotine-dependent animals.

To test these hypotheses, we reanalyzed the previously published whole-brain nicotine withdrawal network (Kimbrough et al., 2021) focusing on the cholinergic regions using hierarchical clustering and graph theory analysis, and investigated the relationship between baseline gene expression levels and Fos reactivity using the whole-brain *in situ* Allen Brain expression database, which contains the regional whole-brain expression of 19,413 genes in the mouse genome (Lein et al., 2007; Davoudian et al., 2023). Contrary to our hypothesis, we found that during nicotine withdrawal, the cholinergic regions did not cluster together in a single module but were instead represented in each of the main modules and organized into two anticorrelated networks that were separated into basal forebrain projecting and brainstem-thalamic-projecting cholinergic regions. Moreover, while mRNA expression of a few nicotinic receptors correlated with Fos activation, we identified a list of >1000 candidate genes and 3 intracellular pathways that may contribute to the reorganization of the whole-brain functional connectome during nicotine withdrawal.

## Materials and Methods

This report includes a reanalysis of a previously acquired and published dataset (Kimbrough et al., 2021) consisting of Fos counts per brain region (175; Extended Data Table 1-1) for two groups of male C57BL/6J mice (60 d old at the start of the experiment), 8 h after removal from minipumps (model 1002, Alzet) that were implanted in the lower back to deliver nicotine (*N* = 5, 24 mg/kg/d) or saline (*N* = 4) for 7 d. This dose was chosen based on previous studies that indicated rewarding effects during use, resulting in withdrawal-like symptoms after the cessation of chronic use (Johnson et al., 2008; Stoker et al., 2012). All brains (*N* = 9, 5 nicotine plus 4 saline) were harvested following perfusion (PBS, followed by 4% formaldehyde), postfixed overnight, immunolabeled for Fos (primary: 1:2000; catalog #226003; Synaptic Systems; and secondary: 1:500; catalog #A31573, Thermo Fisher Scientific; donkey anti-rabbit Alexa Fluor 647), cleared according to the iDISCO+ protocol, imaged using light-sheet microscopy (effective magnification, 1.6×; resolution, 4 × 4  μm; step size, 3 μm; Ultramicroscope II, LaVision BioTec), and analyzed using the ClearMap package (Renier et al., 2016). Three brain regions with low-to-no Fos counts were excluded based on quality control of the original data: the dorsal premammilary nucleus, parabigeminal nucleus, and suprachiasmatic nucleus. These experiments had been conducted in strict adherence to the National Institutes of Health *Guide for the Care and Use of Laboratory Animals*, and approved by The Scripps Research Institute Institutional Animal Care and Use Committee and by the Institutional Animal Care and Use Committee of the University of California. No new experimental procedures were performed for this article. The data were processed similarly as previously published (Kimbrough et al., 2020, 2021) using GraphPad Prism and R (code is available online at https://github.com/George-LabX), as described in more detail below.

### Functional connectome construction

The Fos counts per region obtained from the published dataset, were all increased by 1 and normalized to a log10 value to reduce variability, before calculating Pearson correlations between regions. The matrix was then hierarchically clustered, based on the Euclidean distances calculated from the correlations. Modules were obtained by cutting the clustering dendrogram at half-height.

### Average correlation calculations

Average *R* values were calculated for each treatment (saline or nicotine) within the basal forebrain cholinergic regions (*n* = 3, excluding self-correlations), within the brainstem-thalamic cholinergic regions (*n* = 6, excluding self-correlations), and between both cholinergic subgroups (*n* = 12). Average *R* values were also calculated per treatment for the interaction of all cholinergic regions with the major anatomic groups in the brain. Two-way ANOVA was then performed to examine the effect of treatment condition on the average *R* value for each comparison.

### Network analysis

Networks were analyzed for centrality (degree or betweenness) with the R package iGraph. Participation coefficients were obtained using a customized version of the bctpy Python package (https://github.com/aestrivex/bctpy), derived from the MATLAB implementation of Brain Connectivity Toolbox (Rubinov and Sporns, 2010).

### Analysis of expression data

Structure and gene expression data were extracted from the In Situ Hybridization gene expression database and Allen Brain Atlas (Lein et al., 2007) in Python, as published and described before [AllenSDK (https://doi.org/10.5281/zenodo.3951756); Fulcher and Fornito, 2016; Fulcher et al., 2019; Davoudian et al., 2023] and intersected with the 175 brain regions from the Fos dataset. The expression density of all nicotinic cholinergic receptors in the gene expression atlas was averaged across the experimental sets following centering and scaling per experiment. Next, the correlation of the baseline expression level (percentage of pixels) for every gene in every experiment was correlated with the Fos expression change in withdrawal compared with control (log-fold change) per brain region for every gene. The frontal pole cerebral cortex was excluded as an outlier.

### Reactome analysis

The gene set was analyzed using the Reactome pathway database on https://reactome.org/ by inserting the gene set, projected to human, and analyze without interactors (Joshi-Tope et al., 2005).

### Statistical analysis

Statistical analysis was performed as indicated in GraphPad Prism software or in R Studio. For comparison of the brain states, we used two-way ANOVA with Tukey’s corrected *post hoc* test^a,b^ in Prism or with Mann–Whitney *U* test^c-e^ in R with *p* < 0.05 (Table 1, statistics). For the gene analysis, the significance of the Pearson correlations was evaluated by calculating a *p*-value with the Pearson correlation test in R. We then used the Benjamini–Hochberg (BH) procedure to correct for multiple hypothesis testing and control the false discovery rate (FDR) at a 5% level. This method ensures that, on average, no more than 5% of the statistically significant results are expected to be false positives. By adjusting the raw *p*-values using the BH procedure, we obtained *q*-values for each test. A hypothesis test was considered statistically significant if its *q*-value was less than or equal to the predetermined FDR threshold of 0.05. This approach strikes a balance between controlling the risk of false positives and maintaining adequate statistical power, making it suitable for our analysis involving multiple comparisons.

**Table 1:** Statistical table

	Data structure (Shapiro–Wilk test)	Type of test	Power (95% CI between saline and nicotine mean)
a	Normal	Two-way ANOVA	−0.88 to −0.14
b	Normal	Two-way ANOVA	−0.40 to −0.27
c	Non-normal	Mann–Whitney *U* test	36.00–50.00
d	Non-normal	Mann–Whitney *U* test	−63.03 to −27.80
e	Non-normal	Mann–Whitney *U* test	−0.20 to −0.11

### Data visualization

Graphs were constructed using the R package ggplot2 included in the tidyverse package or the ggpubr package for barplots. Heatmaps were constructed using the R packages gplots and ComplexHeatmap. Networks were visualized by plotting in the R package igraph or using Gephi software. Pathway illustrations were created with BioRender (https://www.biorender.com/). Figures were combined and edited with Adobe Illustrator.

### Data availability

The code described in the article is freely available online at https://github.com/George-LabX. Additional code and data for analyzing the whole-brain expression data are available from https://alexkwanlab.org/data/. The results were obtained by running the code in R Studio on a MacBook Pro.

## Results

### Cholinergic groups are distributed throughout the nicotine withdrawal network

**Figure 1.**
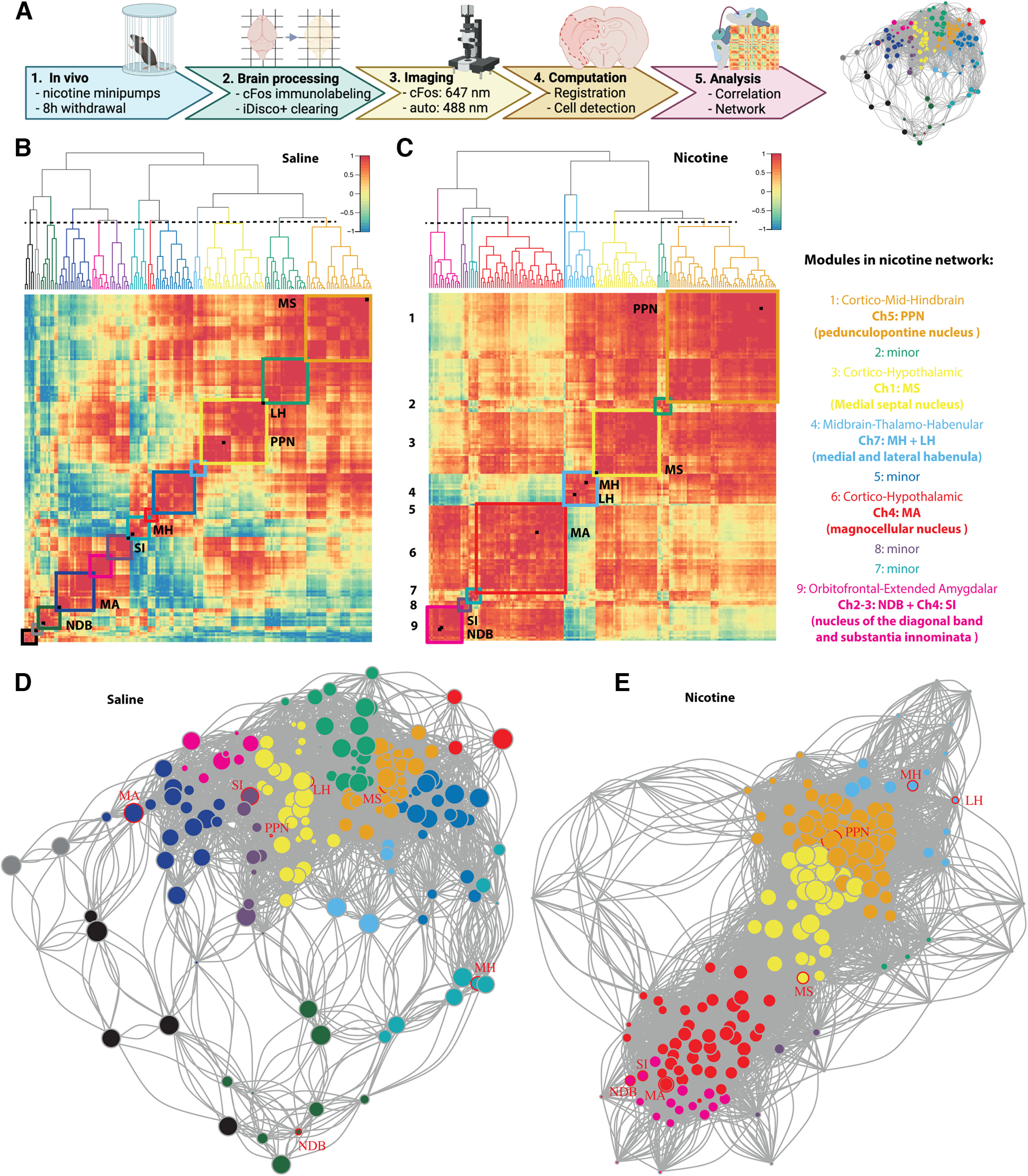
Functional network of nicotine withdrawal in mice. ***A***, Experimental timeline for obtaining the nicotine withdrawal functional connectome: mice were implanted subcutaneously with osmotic minipumps that delivered nicotine for 1 week, 8 h after removal brains were harvested using perfusion (1); the brains were immunolabeled for Fos and cleared using the iDISCO+ protocol (2); next, the brains were imaged using light-sheet imaging at 647 nm for Fos and 488 nm for autofluorescence (3); images were automatically registered to the Allen Brain Atlas, and active cells counted per brain region using ClearMap (4); and the Fos cell counts of each region were correlated per group to obtain distances between the regions to create a network of the brain regions, which could be further analyzed (5). ***B***, ***C***, Hierarchically clustered correlation heatmaps of the resting-state functional connectome under control conditions (saline; ***B***) or nicotine withdrawal (***C***), with 13 and 9 modules, respectively, depicted with colored squares and cholinergic regions labeled as black squares on the diagonal. The order of the brain regions in the heatmaps is available in Extended Data Figure 1-1. ***D***, ***E***, Network graph for saline (***D***) and nicotine withdrawal (***E***) with indication of cholinergic long-range regions (red circles). The node colors represent the different modules and the node size represents the degree (number of connections). A larger image of the network with labeled nodes is available in Extended Data Figures 1-1 and 1-2.

Using the methods summarized in Figure 1*A*, the nicotine and saline control functional connectomes were obtained. Immunolabeling of immediate-early gene expression like c-*fos* captured the neuronal reactivity over a period of 1–2 h during nicotine withdrawal. Automated registration onto an anatomic reference atlas using the ClearMap pipeline (Renier et al., 2016) then allowed for unbiased quantification of neuronal activation throughout the brain. Finally, based on synchronous reactivity between functionally connected brain regions, correlation analysis of the Fos counts allowed calculation of functional distances (Fig. 1*B*,*C*, Extended Data Fig. 1-1) and construction of a whole-brain functional network that can be further analyzed using graph theory (Fig. 1*D*,*E*, thresholded for Pearson correlation > 0.75, Extended Data Figs. 1-2, 1-3). The nicotine withdrawal network consisted of 175 brain regions (nodes) with 4738 functional connections (edges), which was a 50% increase from the saline control that had 3019 functional connections. Hierarchical clustering of the correlation matrices with division of the dendrogram at half-height revealed nine modules, which was a clear decrease from the 13 modules in the control network. The five main nicotine modules included both cortical and subcortical regions, and were named based on the regions with the most significant within-module influence based on the within-module degree *z* score, which measures the intramodule connectivity or relative importance of a region within its own module (Kimbrough et al., 2021). The long-projection cholinergic groups did not cluster together in a single module as originally hypothesized, rather they were spread between the modules. All five main modules contained at least one of the eight main long-projection cholinergic groups (Ch1–8; Fig. 1*D*, circled in red). The largest cortico-mid-hindbrain module contains cholinergic group 5 (PPN). Next, the cortico-hypothalamic module contains cholinergic group 4 (MA). The intermediate cortico-hypothalamic module contains cholinergic group 1 (MS). The smaller orbitofrontal-extended amygdalar module contains cholinergic groups 2 and 3 (NDB), cholinergic group 4 (SI), and the midbrain-thalamo-habenular module cholinergic group 7 (MH) and lateral habenula (LH). The smallest modules did not have any cholinergic regions. Note that cholinergic groups 6 and 8 were omitted as they were too posterior for the imaging.

10.1523/ENEURO.0019-23.2023.f1-1 Figure 1-1 Organization of the regions in the clustered correlation matrices for the saline and nicotine groups (Fig. 1*B*,*C*) Download Figure 1-1, DOCX file.

**Figure 2.**
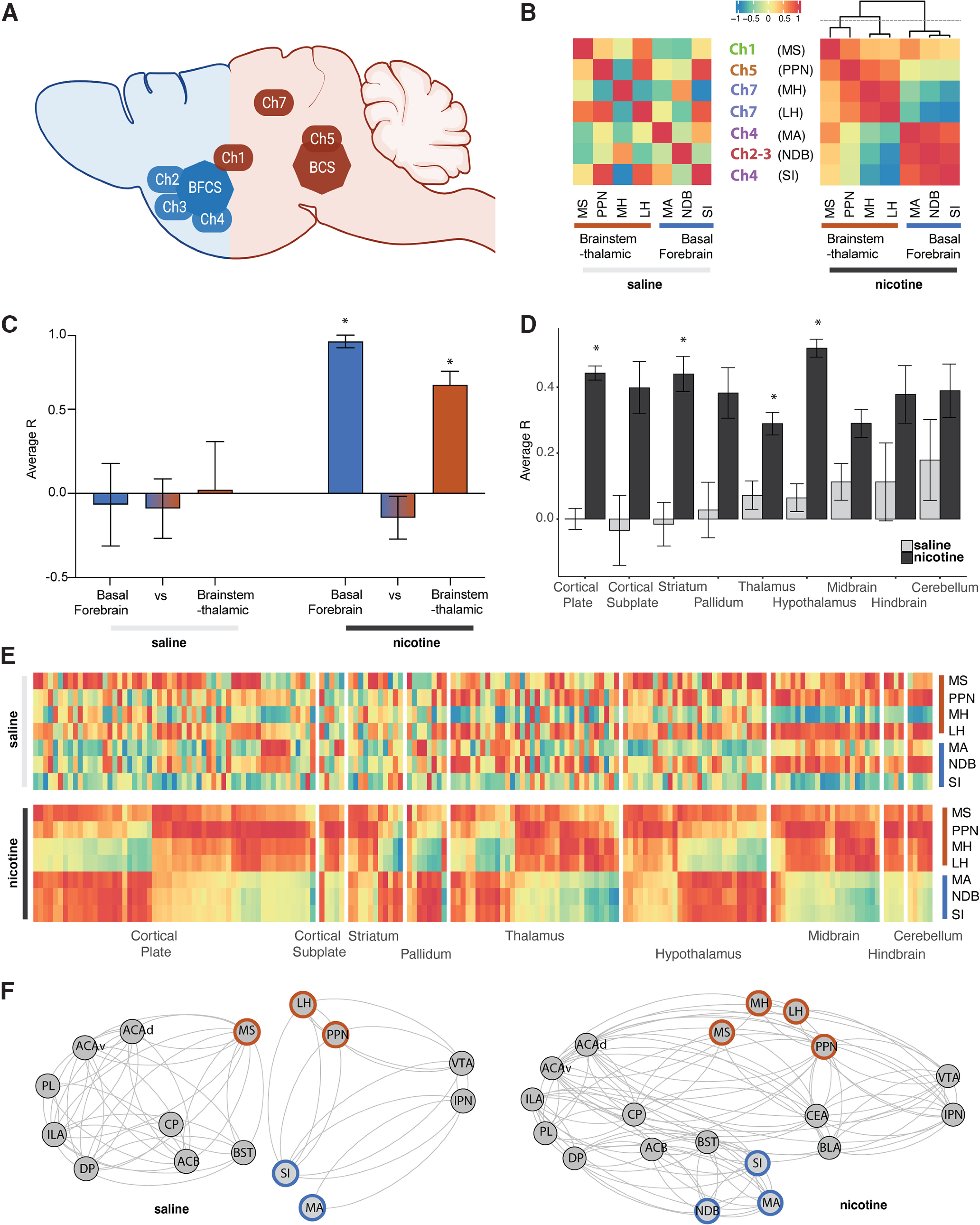
Interactions among the long-range cholinergic groups and with the whole-brain for saline and nicotine. ***A***, Brain schematic showing the localization and circuitry of the long-range cholinergic groups divided in basal forebrain cholinergic system (BFCS; blue) and brainstem-thalamic cholinergic system (BCS; brown), adapted from George et al. (2006). ***B***, Heatmap representation of the correlation of cholinergic long-range groups. ***C***, Average correlation (*R*) between the cholinergic long-range groups within the basal forebrain cholinergic system (blue), brainstem-thalamic cholinergic system (brown), or between (*p < 0.05)^a^. ***D***, Average correlation (*R*) of cholinergic long-range groups with the rest of the brain organized into anatomic groups (*p < 0.05)^b^. ***E***, Heatmap representation of ***D*** with the correlation to all individual regions in the brain (Extended Data Fig. 2-1, order of the regions). ***F***, Integration of the long-range cholinergic groups in representative minimal addiction networks.

10.1523/ENEURO.0019-23.2023.f1-2 Figure 1-2 Detailed view of Figure 1*D* with node labels. Download Figure 1-2, DOCX file.

**Figure 3:**
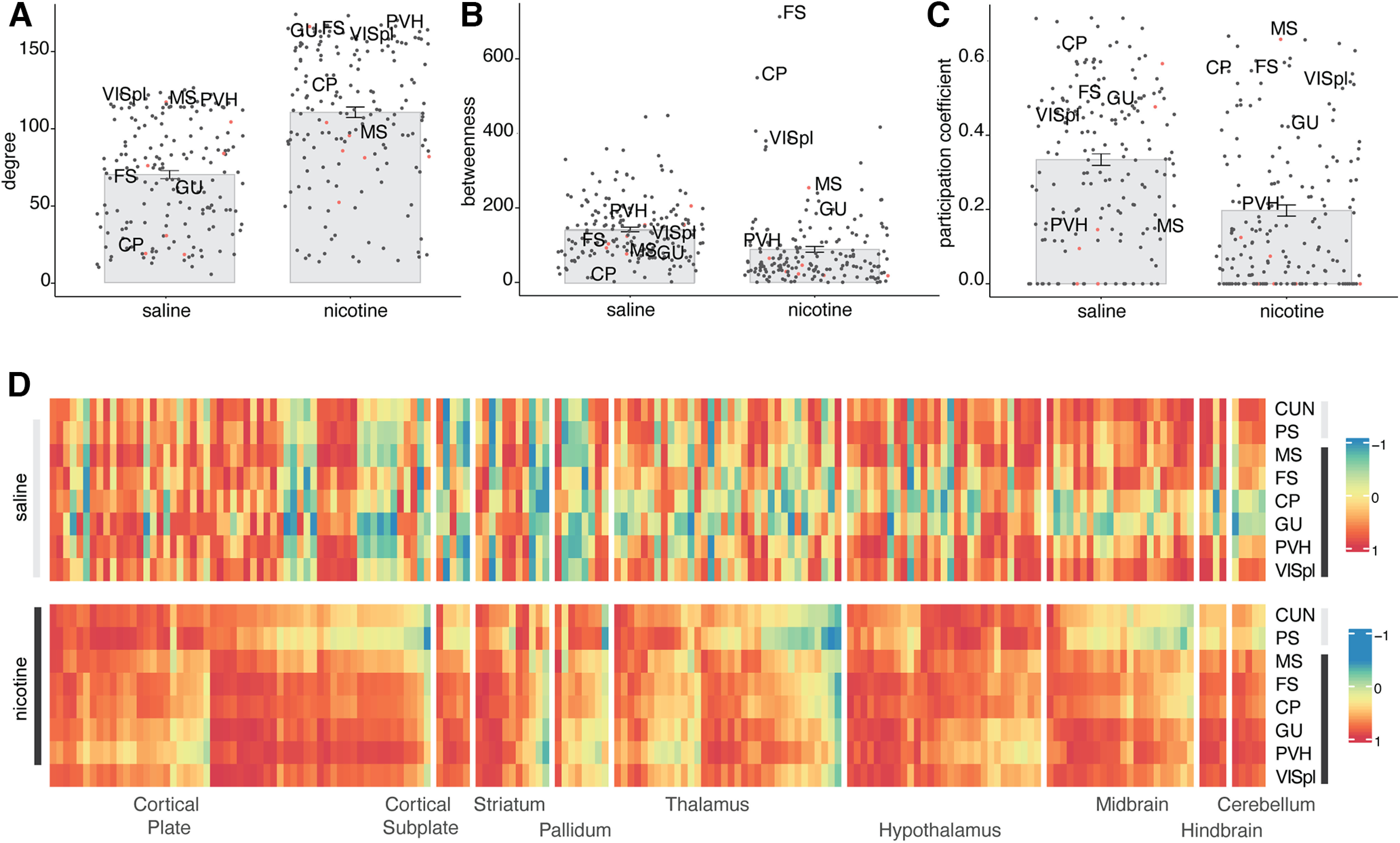
Centrality measurements for hub regions in the saline and nicotine networks. ***A–C***, Degree (*p* < 2.2e-16^c^; ***A***), betweenness (*p* = 2.0e-08^d^; ***B***), and participation coefficient (*p* = 3.5e-9^e^; ***C***), with the long-range cholinergic regions identifiable by a red dot and the hub regions for nicotine withdrawal labeled. ***D***, Heatmap representation of the correlation of the hub regions to all individual regions in the brain (order of the regions same as in Extended Data Fig. 2-1).

10.1523/ENEURO.0019-23.2023.f1-3 Figure 1-3 Detailed view of Figure 1*E* with node labels. Download Figure 1-3, DOCX file.

10.1523/ENEURO.0019-23.2023.tab1-1 Table 1-1 Published Fos counts during nicotine withdrawal and saline control. Download Table 1-1, DOCX file.

### Increased interaction of the long-range cholinergic groups throughout the brain in two subsystems

To investigate the role of the cholinergic regions in the organization of the whole-brain network, we first tested whether the long-range cholinergic groups (Fig. 2*A*) were significantly correlated with each other (Fig. 2*B*). The correlation heatmap focusing on these regions showed the emergence of two anticorrelated cholinergic subsystems during nicotine withdrawal; one in the basal forebrain consisting of MA, NDB, and SI, and one in the brainstem-thalamic area consisting of PPN, MH, and LH, with the MS in between. Under control conditions (saline), there was no such organization. The average correlation between the cholinergic regions within the basal forebrain or within the brainstem-thalamic network was significantly higher in the nicotine group compared with the saline group (two-way ANOVA: *F*_(1,36)_ = 7.85, *p* = 0.008), with a significant difference between regions (two-way ANOVA: *F*_(2,36)_ = 4.62, *p* = 0.016), and a significant interaction (two-way ANOVA: *F*_(2,36)_ = 3.36, *p* = 0.046; Fig. 2*C*)^a^. *Post hoc* analysis confirmed that under nicotine withdrawal the average correlation within the subgroups was significantly higher than their interaction (*p* < 0.04).

10.1523/ENEURO.0019-23.2023.f2-1 Figure 2-1 Organization of the regions in the correlation matrix separated by anatomical groups (Figs. 2*E*, 3*D*). Download Figure 2-1, DOCX file.

We then looked at how these long-range cholinergic groups correlated with the different anatomic groups throughout the brain using hierarchical clustering within the main anatomic structures (cortical plate, cortical subplate, striatum, pallidum, thalamus, hypothalamus, midbrain, hindbrain, or cerebellum; Fig. 2*D*,*E*). There was a significant increase in correlation throughout the brain in the nicotine group compared with the saline group (two-way ANOVA: *F*_(1,2082)_ = 100.7, *p* < 0.0001), without significant difference between regions (two-way ANOVA: *F*_(8,2082)_ = 1.32, *p* = 0.23), but with significant interaction (two-way ANOVA: *F*_(8,2082)_ = 3.21, *p* = 0.0012)^b^. In line with the overall increase in correlation, the *post hoc* test showed an increase in the correlation of the cholinergic groups with the cortical plate (*p* < 0.0001), striatum (*p* < 0.0001), thalamus (*p* = 0.0035), and hypothalamus (*p* < 0.0001).

Finally, we looked at the interaction of the long-range cholinergic regions with brain regions that have been shown to be critical to nicotine addiction and nicotine withdrawal, including the anterior cingulate area (ACA; Hong et al., 2009; Wang et al., 2019; Abulseoud et al., 2020), infralimbic area (ILA; George and Koob, 2010; Huang et al., 2015; Kutlu et al., 2016), prelimbic area (PL; George and Koob, 2010; Semenova et al., 2018), dorsal peduncular area (DP; George and Koob, 2010), caudoputamen (CP; Muskens et al., 2012; Huang et al., 2015), nucleus accumbens (ACB; Rada et al., 2001; Schmidt et al., 2001; Huang et al., 2015), bed nucleus of the stria terminalis (BST; Reisiger et al., 2014; Qi et al., 2016), basolateral amygdala (BLA; Bergstrom et al., 2010; Sharp, 2019), central amygdala (CEA; Baiamonte et al., 2014; Huang et al., 2015; Funk et al., 2016), ventral tegmental area (VTA; Grieder et al., 2014; Huang et al., 2015; Wills and Kenny, 2021), and interpeduncular nucleus (IPN; Molas et al., 2017; Wills and Kenny, 2021; Klenowski et al., 2022). Here too, the minimal networks showed an overall increased functional connectivity during nicotine withdrawal, particularly among the cortex, subcortical regions, and key cholinergic regions including cholinergic groups 2, 3, 4, and 7 (PPN, MH, LH, SI, MA, and NDB), which were separated in basal forebrain and brainstem-thalamic groups (Fig. 2*F*).

### Non-long-range cholinergic regions function as connector hubs in the nicotine network

To better understand the role of the cholinergic groups within the whole-brain network and to validate their role as hub regions or identify others, we calculated the network centrality measures: degree (number of connections a region has) and betweenness (number of shortest paths between two regions that go through a region). Nicotine withdrawal significantly increased the average degree (*p* < 2.2e-16^c^; Fig. 3*A*) and decreased the average betweenness of the network (*p* = 2.0e-08^d^; Fig. 3*B*). Regions that were in the top 20 of both degree and betweenness were considered hub regions (Wheeler et al., 2013). For the saline network, two hubs with both high degree and betweenness were identified: the hypothalamic parastrial area (PS) and the midbrain cuneiform nucleus (CUN). For the nicotine network, the following four hubs with those criteria were identified: the fundus of the striatum (FS), paraventricular hypothalamic nucleus (PVH), gustatory areas (GUs), and posterolateral visual areas (VISpls). The fundus of the striatum and caudoputamen stood out for having significantly higher betweenness scores than other regions, thus having a central role in the network involving shortest paths during nicotine withdrawal.

Because the networks are modular, an important role of hub regions is to act as connectors between modules, which is captured through a high participation coefficient that measures the intermodule connectivity or the extent to which a region connects to multiple other modules. Regions with a high participation coefficient were therefore also considered as hubs. Nicotine withdrawal significantly decreased the participation coefficient (*p* = 3.5e-9^e^; Fig. 3*C*). The cholinergic group 1 MS had the highest participation coefficient of the network and thus functions as a top connector between the network modules. The fundus of the striatum and caudoputamen hubs also scored high for this measure. The central role of these regions in the network is confirmed by looking at their correlation with all brain regions (Fig. 3*D*), which showed strongly increased correlation during nicotine withdrawal.

### Identification of novel gene targets that correlates with brain-wide Fos activation

**Figure 4.**
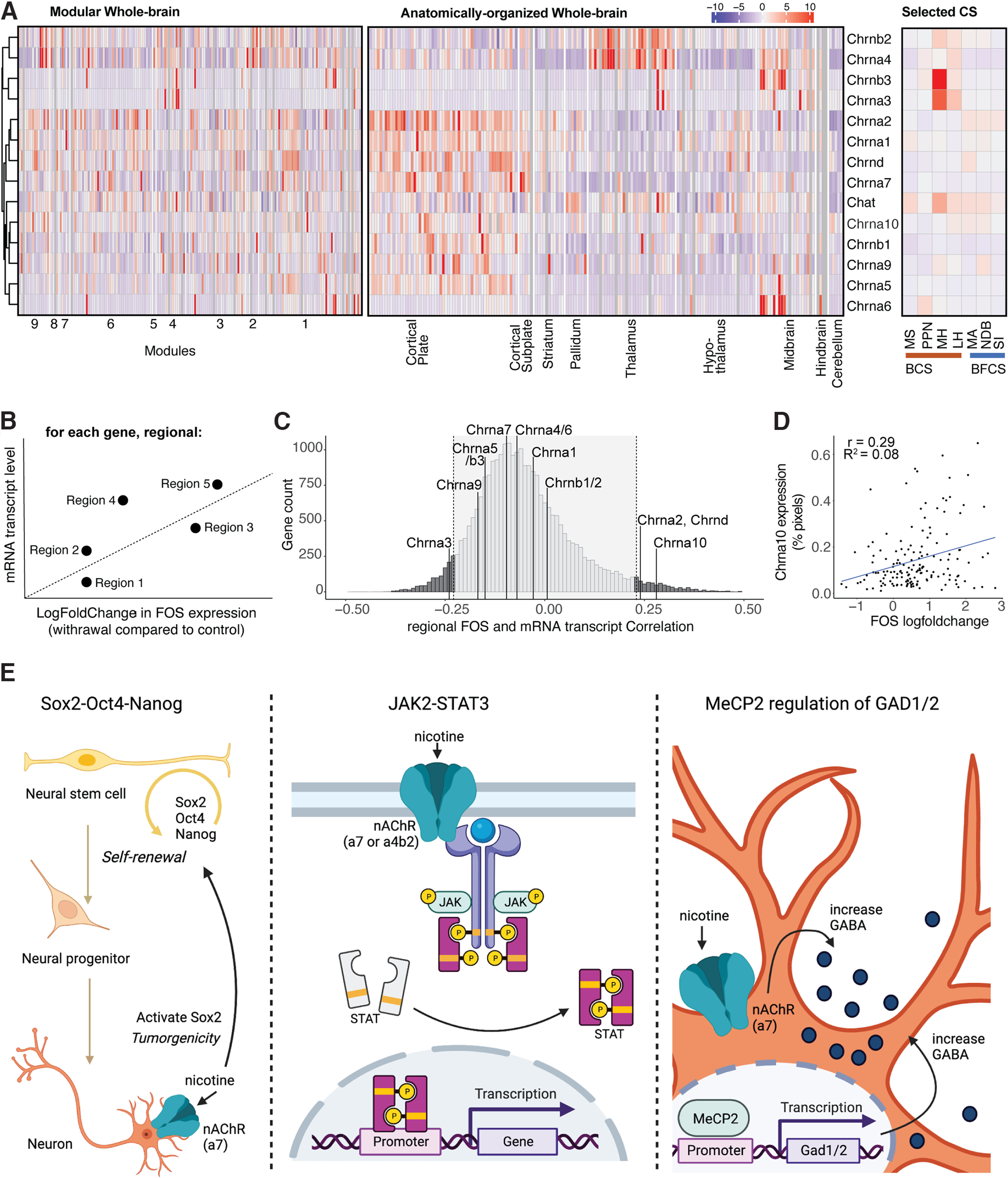
Whole-brain expression distribution of cholinergic receptor subunits, cholinergic transferase, and other proteins. ***A***, Expression density throughout the whole brain hierarchically clustered by row and split into the modules of the nicotine-withdrawal network (left; Fig. 1*C*, ordering of regions; Extended Data Fig. 1-1, list), split into anatomic groups (middle; Fig. 2*E*, ordering of regions; Extended Data Fig. 2-1, list) and the selected long-range cholinergic groups (right; Fig. 2*B*, ordering of the regions). ***B***, Schematic diagram of the correlation analysis for each gene between basal mRNA expression level and nicotine withdrawal-induced Fos transcriptional change in each region. ***C***, Histogram of the number of genes (count) for all found correlations. ***D***, Example correlation graph for the most correlated cholinergic-related gene Chrna10. ***E***, Schematic representations of the pathways identified by the Reactome analysis for the identified significantly correlated genes: Oct4, Sox2, and Nanog activating genes related to proliferation (left), gene expression by JAK-STAT signaling (middle), and the MeCP2 pathway for regulating the transcription for genes involved in GABA signaling through GAD1 and GAD2 (right), with the potential involvement of nicotine.

To examine the contribution of the regional expression level of cholinergic-related genes like the nAChRs to the organization of the functional connectome, the basal expression level of *Chrna1-10*, *Chrnb1-3*, *Chrnd*, and *ChAT* throughout the brain was extracted from the *in situ* Allen Brain database (Lein et al., 2007) and examined. While *Chrna1*, *Chrnb1*, and *Chrnd* are generally considered muscle-type subunits, expression in the brain has been observed (Aishah et al., 2017). No clear pattern could be observed differentiating expression in the different modules of the functional connectome (Fig. 4*A*, left; expression within the long-range cholinergic groups is highlighted on the right). When organizing the brain regions in anatomic order on the other hand, expression patterns could be observed with *Chrna4* and *Chrnab2* being expressed mostly in the thalamus; *Chrna3*, *Chrna6*, and *Chrnb3* mostly in the midbrain; and *Chrna1*, *Chrna2*, *Chrna7*, *Chrna9*, *Chrna10*, *Chrnb1*, and *Chrnd* mostly in the cortical plate (Fig. 4*A*, middle).

10.1523/ENEURO.0019-23.2023.tab4-1 Table 4-1 List of significantly correlated genes (Fig. 4*C*, FDR, 5%). Download Table 4-1, DOCX file.

Next, we wanted to compare the contribution of these cholinergic-related genes to the changes in whole-brain Fos activation and compare it with the other 19,413 genes of which the *in situ* Allen Brain database contains the region-specific gene expression (Lein et al., 2007). For every gene, we looked at the correlation between the baseline gene expression level (percentage of pixels) and the Fos expression change in nicotine withdrawal versus saline control (log-fold change) for every region (Davoudian et al., 2023; Fig. 4*B*). Significance was obtained for genes with a correlation coefficient higher than |0.23| (false discovery rate, <5%; Fig. 4*C*), which included *Chrna2*, *Chrna3*, *Chrna10* (Fig. 4*D*), and *Chrnd*. The expression of the other cholinergic-related genes did not significantly correlate with the increased induction of Fos expression during nicotine withdrawal. However, we identified 1755 genes that were significantly correlated with Fos expression during withdrawal (false discovery rate, < 5%; Extended Data Table 4-1).

To investigate the obtained gene list, it was inserted into Reactome, the free, open-source, curated, and peer-reviewed pathway database (Joshi-Tope et al., 2005), which returned the following three top hits: (1) octamer-binding transcription factor 4 (OCT4), sex-determining region Y-box 2 (SOX2), and nanog homeobox (NANOG) activate genes related to proliferation (*p* = 5.12e-3); (2) gene and protein expression by janus kinase (JAK), signal transducer and activator of transcription (STAT) signaling after Interleukin-12 stimulation (*p* = 1.08e–2), and methyl CpG binding protein 2 (MeCP2) regulation of transcription of genes involved in GABA signaling (*p* = 6.91e–3; Fig. 4*E*).

## Discussion

This work follows up on the published established whole-brain nicotine withdrawal network obtained through single-cell whole-brain imaging of the immediate early gene c-Fos compared with controls (Kimbrough et al., 2021), focusing on the long-range cholinergic regions to help interpret and understand specific functional connectome changes. Contrary to our hypothesis, the well defined long-range cholinergic groups (Ch1–7) were not found to cluster together, but rather were distributed throughout the nicotine withdrawal network. Cholinergic regions showed increased functional connectivity with all regions of the brain through two anticorrelated subnetworks separated into basal forebrain-projecting and brainstem-thalamic-projecting cholinergic regions, validating a long-standing hypothesis of the organization of the brain cholinergic systems. Most of the cholinergic-related genes were found to have whole-brain expression profiles that correlated poorly with the nicotine withdrawal-induced Fos changes except for *Chrna2*, *Chrna3*, *Chrna10*, and *Chrnd* mRNA. Finally, we identified a list of >1700 genes for which the baseline expression correlated significantly with the altered brain reactivity in the nicotine withdrawal state and identified cellular pathways that may contribute to neuronal activation during nicotine withdrawal.

This report demonstrates that each of the main modules in the nicotine withdrawal network includes at least one of the well defined long-range cholinergic groups (Ch1–7), and that the localization of each group within each module was consistent with known anatomic and functional connections for these groups (Fig. 1). Cholinergic group 1 is the primary cholinergic input to the hippocampus (Teles-Grilo Ruivo and Mellor, 2013; Müller and Remy, 2018) and was found in the cortico-hypothalamus module. Cholinergic group 5 is the primary cholinergic input for the brainstem (Grofova and Keane, 1991; Mena-Segovia and Bolam, 2017) and was part of the cortico-mid-hindbrain module. Cholinergic groups 2, 3, and 4 are the primary projections to the isocortex, striatum, and amygdala (Mesulam et al., 1983; Luiten et al., 1987; Alheid and Heimer, 1988) and were found in the orbitofrontal-extended amygdalar and cortico-hypothalamic modules. Finally, cholinergic group 7 projects to the brainstem and was found in the midbrain-thalamo-habenular module. Cholinergic neurons have been described to act and project globally rather than modularly, which helps in communication throughout the whole brain (Mesulam et al., 1983; Woolf, 1991).

Nicotine withdrawal had strong effects on the functional connectome. First, the functional connectivity was increased between the long-range cholinergic regions and the rest of the brain (Fig. 2), particularly with the regions that had lower functional connectivity under control conditions such as the cortical plate, striatum, thalamus, and hypothalamus. The increased synchronization between the long-range cholinergic regions and the rest of the brain may contribute to the synchronization of Fos activity throughout the brain, resulting in decreased modularity (Kimbrough et al., 2021). A possible mechanism underlying this brain-wide synchronization is a global increase in acetylcholine release during withdrawal (Rada et al., 2001; Carcoba et al., 2014), leading to the activation of nAChRs, intracellular cation influx, and activation of multiple intracellular cascades activating c-Fos transcription (Merlo Pich et al., 1999; Hu et al., 2002; Changeux, 2010; Mizuno et al., 2015). These results are in line with human fMRI data, where increases in resting-state connectivity during nicotine withdrawal have also been observed (Fedota and Stein, 2015). Moreover, increased local connectivity within specific network nodes correlate with subjective measures of nicotine craving and measures of nicotine dependence (Claus et al., 2013; Janes et al., 2014; Moran-Santa Maria et al., 2015).

Second, nicotine withdrawal caused a functional reorganization of the long-range cholinergic network composed of the MA, NDB, and SI on one side, and the MS, PPN, MH, and LH on the other side, which are correlated within, anticorrelated between, and connected to mostly nonoverlapping regions in the brain. Also, when looking at a minimal network containing the long-range cholinergic regions and key regions known to be involved in addiction, the same findings were illustrated, as follows: (1) increased functional connectivity among the cholinergic groups 2, 3, 4, and 7 and the anterior cingulate, infralimbic, prelimbic, dorsal peduncular, ventral tegmental area, caudoputamen, nucleus accumbens, basolateral and central amygdala, bed nucleus of the stria terminalis, and interpeduncular nucleus; and (2) increased subdivision of the circuitry in basal forebrain and brainstem-thalamic cholinergic systems. Indeed, several cholinergic regions (MH, MA, NDB) were not incorporated in this minimal network under control conditions. The organization of a basal forebrain and a brainstem cholinergic system resembles the original anatomic descriptions of the basal forebrain and brainstem cholinergic systems by Mesulam et al. (1983), Woolf (1991), and George et al. (2006), which not only validates the approach of single-cell whole-brain imaging for functional connectome analysis, but has profound implications from a theoretical point of view. Indeed, it suggests that the different cholinergic regions throughout the brain are not independent from each other, but instead are functionally connected through two opposite systems: a basal forebrain cholinergic system and a brainstem cholinergic system. Nicotine withdrawal then emerges with the dysregulation of these two systems that become anticorrelated. Whether one cholinergic system inhibits the other or whether they are anticorrelated though the action of a third system remains to be tested.

Third, the central hubs of the network changed. In the saline control network, the CUN and PS acted as hub regions with high degree and betweenness, measures of network centrality (Fig. 3; Wheeler et al., 2013). Following nicotine withdrawal, the fundus of the striatum and caudoputamen were identified as hub regions with the high network centrality measures degree and betweenness. The long-range cholinergic region MS (cholinergic group 1), which is the main input of the hippocampus and has been associated with the anxiogenic effects of nicotine (Zarrindast et al., 2013) had a high participation coefficient and therefore also participated as a hub, especially in the connection between the different modules. Its connector role between the basal forebrain and brainstem cholinergic systems was already illustrated in Figure 2*B*. While the fundus of the striatum is typically not recognized as a long-range cholinergic region, it is a transition zone between the ventral part of the caudoputamen and the substantia innominate (cholinergic group 4) that expresses high levels of acetylcholine esterase and where dopamine release is under a particular tight cholinergic control (O’Connor et al., 1995). The caudoputamen is the brain region with the highest basal acetylcholine level because of a dense cholinergic arborization originating from cholinergic interneurons (Zhou et al., 2002; Gonzales and Smith, 2015; Abudukeyoumu et al., 2019). Caudoputamen cholinergic interneurons are critical to dopamine release, reinforcement learning and the formation of habit (Knowlton et al., 1996; Matsumoto et al., 1999; Kitabatake et al., 2003). These hub regions all play central roles in orchestrating the negative emotional state under nicotine withdrawal and were all found together in the intermediate-size module 3 (Extended Data Figs. 1-1, 1-3). The medial septum—fundus of the striatum—caudoputamen module might thus function as the main nicotine-responsive module that orchestrates the whole-brain response.

All nicotinic receptor genes, except the muscle-type CHRNB1, including eight genomic regions containing 11 neuronal CHRN genes and 3 genomic regions containing 4 muscle-type CHRN genes, have been significantly associated with nicotine dependence and/or alcohol dependence (Zuo et al., 2016). Analysis of the correlation between baseline mRNA expression of the nAChRs in all brain regions with the withdrawal-induced change in Fos expression (Fig. 4*B*) showed significant correlation for *Chrna2*, *Chrna3*, *Chrna10*, and *Chrnd* (Fig. 4*C*,*D*). *Chrna2* has been identified in human genome-wide association studies (GWASs) in association with smoking-related behaviors, like smoking status, smoking initiation, cigarettes smoked per day, and smoking cessation (Liu et al., 2019; Xu et al., 2020). *Chrna3* is part of a locus on chromosome 15q25 with *Chrna5* and *Chrnb4*, which has also been identified in human GWASs to be associated with smoking-related behaviors and nicotine dependence. One single nucleotide polymorphism is specifically localized in *Chrna3* (Spitz et al., 2008; Liu et al., 2010). *Chrna10* was identified through linkage analysis in sibling pairs for nicotine withdrawal (Pergadia et al., 2009) and was found, together with *Chrnd*, to increase the risk for nicotine dependence in an African American population subset (Saccone et al., 2010). It is important to note that these correlations were obtained using baseline gene expression with no exposure to nicotine, suggesting that these genes may be predisposing factors to nicotine dependence. However, further studies are required to examine the correlation between withdrawal-induced gene expression and withdrawal-induced Fos activity. Looking at the mRNA expression was a first attempt to link nAChRs levels to Fos activation. A limitation is that mRNA expression does not necessarily correlate with protein levels or even functional activity of the protein; therefore, while we observed significant correlations between some nAChRs and differential Fos expression, it is possible that negative results for other subunits may be because of a lack of correlation between mRNA levels and protein levels, for instance because of post-transcriptional events (Mousavi et al., 2003).

Other study limitations are associated with the dataset. Fos is inherently not a great marker for neuronal inhibition and therefore poorly detects negative correlations between brain regions resulting from activation of inhibitory neurons. The use of the recently identified marker for neuronal inhibition could reduce this gap in future studies (Yang et al., 2023). The dataset also has a relatively low sample size. In our experience, the comparison of groups of brain regions that are correlated versus regions that are not correlated (among the ≥175 brain regions) is associated with large effect sizes (Cohen *d*, >1.8) and requires only *N* = 4–5/group for significant findings, which is in line with what has been reported previously (Wheeler et al., 2013; Orsini et al., 2018; Kimbrough et al., 2020, 2021). A higher sample size would increase the ability to detect effects that have moderate to small size and further dissect the different brain networks contributing to nicotine dependence. Finally, both the original whole-brain reactivity (Kimbrough et al., 2021) and whole-brain gene expression (Lein et al., 2007) studies only incorporated male subjects in their study and database precluding any analysis of sex differences. Follow-up studies are needed to evaluate whether these effects also exist in females.

We then extended the gene analysis to include the mRNA transcript levels of all 19,413 genes of the *in situ* hybridization gene expression Allen Brain Atlas database, which resulted in a list of 1755 genes that had significant correlations between their expression and the nicotine withdrawal-induced Fos changes. Through pathway analysis (Joshi-Tope et al., 2005), we identified potentially promising genes and pathways that may contribute to the Fos expression during nicotine withdrawal. The first pathway contained the transcription factors *Sox2*, *Oct4*, and *Nanog*, which are highly expressed in proliferative adult neurogenesis precursor cells in discrete brain regions (Suh et al., 2007; Bennett et al., 2009; Ahlfeld et al., 2017; Stevanovic et al., 2021) that are associated with Fos expression (Velazquez et al., 2015) and have been reported to be affected by nicotine (Brooks and Henderson, 2021). The second pathway was part of the immune response by JAK2-STAT3, which has been shown to be activated by nicotine through complex formation with Chrna7 or Crna4/Chrnab2 to provide neuroprotective effects (Shaw et al., 2002; Wang et al., 2022). The third pathway brings up another very relevant transcription factor, MeCP2, which is mutated in the neurodevelopmental disorder Rett syndrome (Amir et al., 1999). MeCP2 knock-out mice have reduced ChAT-positive cells, which reduced Chrna4 and Chrna6 expression, an attenuated behavior response to nicotine (Leung et al., 2017), dysfunctional reduced GABA signaling (Chao et al., 2010), and some reversed deficits following nicotine administration (Zhang et al., 2016). MeCP2 has been shown to modulate the effects of drugs of abuse in preclinical models (Deng et al., 2010; Im et al., 2010; Repunte-Canonigo et al., 2014; Xu et al., 2021). GABA signaling, regulated by MeCP2 through GAD1 and GAD2 that were both in the gene list, has a well established role in nicotine dependence and withdrawal (Markou, 2008; D’Souza and Markou, 2013; Klenowski et al., 2022). Despite relatively low significance for these pathways (loss of significance with correction for multiple comparisons; false discovery rate, 80%), the top three identified pathways have been shown to be affected by or associated with nicotine, providing validity to this exploratory approach, which might be a way to further process and investigate the obtained whole-brain functional connectome datasets. Finally, these results demonstrate the power of using single-cell whole-brain imaging combined with whole-brain transcriptomics to identify new brain regions, new gene targets, and new cellular pathways that may contribute to nicotine dependence and substance use disorder in general.

In conclusion, these results demonstrate that cholinergic regions increased functional connectivity with the rest of the brain through two anticorrelated subnetworks separated into basal forebrain projecting and brainstem-thalamic-projecting cholinergic regions. The expression level of *Chrna2*, *Chrna3*, *Chrna10*, and *Chrnd* mRNA throughout the brain was correlated with the nicotine withdrawal-induced Fos changes. Finally, we have identified a list of over ∼1700 genes for which the baseline expression correlates significantly with the altered brain reactivity in the nicotine withdrawal state and identified cellular pathways that may contribute to neuronal activation during nicotine withdrawal.

## Synthesis

Reviewing Editor: Leandro Vendruscolo, National Institute on Drug Abuse Decisions are customarily a result of the Reviewing Editor and the peer reviewers coming together and discussing their recommendations until a consensus is reached. When revisions are invited, a fact-based synthesis statement explaining their decision and outlining what is needed to prepare a revision will be listed below. The following reviewer(s) agreed to reveal their identity: Amy Janes.

N/Z

## Author Response

Reviewer 1:

This manuscript describes reanalysis of a previously published data set that counted FOS from throughout the brain of mice, and then correlates this with existing published datasets of CHRN genes. The hypothesis are not well developed and there is not sufficient information around the experimental design and procedures to accurately assess the study. Because of this it is difficult to understand the implications of these findings.

We are very appreciative of this feedback to help us improve the manuscript, we thoroughly expanded on the description of the previous work, and included additional information and descriptions to clarify the hypotheses and illustrate the implications of the findings.

Intro:

1. Please expand what this means “which led to a significant decrease in whole-brain modularity, a reduction of total hub brain regions, and a shift from a cortical- to subcortical-driven network”.

We have expanded this statement to better explain this point (on page 2): “This increased correlation between the brain regions caused the regions to cluster closer together and thus led to a significant decrease in whole-brain modularity, as measured by the decrease in the number of main clusters (after setting the hierarchical cluster dendrograms at half height). Increased functional connectivity throughout the network also resulted in a reduction of brain regions identified as hubs. Hub regions are regions with the highest intramodular and intermodular connectivity as measured using graph theory (participation coefficient, within-module degree).

These hub regions are hypothesized to be the biggest drivers of neuronal activity within the network. For instance, during nicotine withdrawal, the main hub regions shifted from cortical (e.g. sensory, motor cortices) to subcortical (e.g. amygdalar, thalamic, hypothalamic, and midbrain) regions.

2. Clarify what this hypothesis is based on “We hypothesized that nicotine withdrawal would result in synchronization of all cholinergic regions within the largest functional module which would explain the hyperconnectivity and decreased modularity observed during withdrawal”.

The hypothesis is now edited to read (on page 3): “We hypothesized that following chronic nicotine administration, most cholinergic regions that are rich in nicotinic receptors would have a synchronized correlated activity due in part to the brain-wide upregulation of nicotinic receptors (Govind et al., 2009; Fowler et al., 2020) and the increase in cholinergic transmission during nicotine withdrawal (Carcoba et al., 2014). The increased correlation would lead to larger modules and decreased modularity.”

3. Clarify why regional expression of cholinergic genes would predict regions with high FOS expression during nicotine withdrawal and the basis for the assumption that nAChR mRNA would reflect functional changes in cholinergic activity (e.g. protein-based changes in functional nAChRs) i.e. how would changes in mRNA link to processes such as desensitization and regulatory changes. This subhypothesis is now edited to read (on page 3): “Furthermore, since cholinergic receptor signaling is critical for nicotine-induced FOS activation (Pang et al., 2016; Simmons et al., 2016), a subhypothesis was that the regional expression level of cholinergic-related genes would be correlated to regional differential FOS expression under withdrawal in nicotine dependent animals.” Note that we also changed the word “predict” to “correlate” here and in the abstract to avoid the strong connotation associated with “predict”, as well as the words “FOS expression” to “differential FOS expression” to better reflect the dependent measure used (differential expression between withdrawal and control animals). We fully agree with the reviewer that mRNA levels do not necessarily correlate with protein levels or even functional activity and have added this statement in the discussion (on page 15): “Looking at the mRNA expression was a first attempt to link nAChRs levels to Fos activation. A limitation is that mRNA expression does not necessarily correlate with protein levels or even 2 functional activity of the protein, therefore, while we observed significant correlations between some nAChRs and differential Fos expression, it is possible that negative results for other subunits may be due to a lack of correlation between mRNA levels and protein levels for instance due to posttranscriptional events (Mousavi et al., 2003).”

4. Please clarify the selection of Fos and how this reflects hyperconnectivity rather than co-ordinated activation which may include inhibitory neuronal activation. Fos was selected as it is the most used marker of neuronal reactivity, which allows for comparison with a lot of published work, including our own. This was added to the introduction (on page 1): “the expression of the immediate-early gene FOS, a marker of neuronal reactivity”. Because of its low basal expression, Fos is not a good marker to detect neuronal inhibition and negative correlations between brain regions. For instance, in this dataset the number of negative correlations are several orders of magnitude lower than positive correlations. This is also why the field typically does not analyze negative correlations as they represent a minority and are mathematically difficult to compute and interpret.

5. Please clarify the rationale for correlating FOS activity in mice undergoing nicotine treatment with CHRN genes of non-treated mice. The rational is that since cholinergic receptors are critical for nicotine-induced FOS activation (Pang et al., 2016; Simmons et al., 2016), the higher the baseline expression of nicotinic receptors, the more sensitive the brain regions will be to nicotine and nicotine withdrawal. We are very well aware that using mRNA expression at baseline compared to withdrawal is an important limitation of the analysis as it is very likely that nicotine dependence per se will change nAChR mRNA, however, there is no dataset currently available that has brain-wide transcriptomic data in nicotine dependent mice. Looking at the baseline expression is a first attempt to link nAChRs levels to Fos activation, and indeed we found that the baseline levels of several nAChRs expression were correlated with Fos reactivity. Further studies should investigate if this relation is increased or decreased using gene expression of nicotine dependent subjects. This statement was clarified through the changes made in response to comment 1.

Methods

6. Please include justification of selecting only male animals when nicotine dependence impacts both males and females.

Unfortunately, we cannot change the design of the previous study, but we can address this limitation in the discussion. Note that the Allen Brain ISH database only includes male mice in its database which facilitates the interpretation of our analysis since both studies used male mice. Such statement was added in the discussion (on page 15): “Finally, another limitation is that both the original whole-brain reactivity (Kimbrough et al., 2021) and whole-brain gene expression (Lein et al., 2007) studies only incorporated male subjects in their study and database precluding any analysis of sex differences. Follow up studies are needed to evaluate if these effects also exist in females.”

7. As a stand alone publication, please include details of the experimental procedure. The methods don’t state the species or treatment procedures. Justification of procedures isn’t included. Additional details are now provided in the materials and methods section (on page 4): “two groups of male C57BL/6J mice (60 days old at the start of the experiment), 8h after removal from minipumps (Alzet; model 1002) that were implanted in the lower back to deliver nicotine (N=5, 24 mg/kg/d) or saline (N=4) for 7 days. This dose was chosen based on previous studies that indicated rewarding effects during use, resulting in withdrawal-like symptoms after the cessation of chronic use (Johnson et al., 2008; Stoker et al., 2012). The brains were harvested following perfusion (phosphate buffered saline, followed by 4% formaldehyde), postfixed overnight immunolabelled for FOS (primary: 1:2000, Synaptic Systems catalog #226003 and secondary: 1:500; Invitrogen, catalog #A31573, donkey anti-rabbit Alexa Fluor 647), cleared according to the iDISCO+ protocol, imaged using light-sheet microscopy (Ultramicroscope II; LaVision Biotec at 1.6x effective magnification at resolution of 3 4 x 4 μm and a 3 μm step size), and analyzed using the ClearMap package (Renier et al., 2016). Three brain regions with low-to-no FOS counts were excluded based on quality control of the original data: the dorsal premammilary nucleus, parabigeminal nucleus, and suprachiasmatic nucleus. These experiments had been conducted in strict adherence to the National Institutes of Health Guide for the Care and Use of Laboratory Animals and approved by The Scripps Research Institute Institutional Animal Care and Use Committee and by the Institutional Animal Care and Use Committee of the University of California. No new experimental procedures were performed for this manuscript.”

8. There should be a clear description of how adjustments for multiple comparisons were made. A FDR of 5% is generous considering the number of comparisons made, please include justification. Multiple comparison correction, like Bonferroni for instance, may be too strict for the number of comparisons that we make, therefore, most people in the field control false positives by calculating the false discovery rate (FDR), using the Benjamini-Hochberg principle. This approach allows for a more balanced trade-off between false positives and false negatives. Using this principle, a q-value of 0.05% or FDR of 5% (5% of significant results will be false positives) is the norm, which in our case is 88 genes out of 1755 significant genes from a total of 19,413 genes, and seems especially appropriate for the flexibility we want to include, given we are looking at mild correlation with baseline expression of genes. The Bonferroni corrected p-values were now also added to the Extended Data Table 4-1 for comparison, completeness, and potential further exploration. These results show that Bonferroni p < 0.05 corresponds to 94 genes (with no false positives) at q < 0.0005. An intermediate definition with FDR of 1% (Bonferroni p = 1) results in a list of 591 genes (with 6 false positives). These genes can be selected from the extended data table 4-1, which is organized according to FDR and when put into Reactome, we still find: 1) Octamer-binding transcription factor 4 (OCT4), sex determining region Y-box 2 (SOX2), nanog homeobox (NANOG) activate genes related to proliferation though not as the top hit and with lower p=1.28E-2. Additional explanation was added in the statistical analysis section of the materials and methods (on page 6): “Results were considered significant with p < 0.05 (limit 5% in tests as false positives). In cases of multiple comparisons, p-values were corrected using Benjamini-Hochberg (BH) false discovery rate (FDR) with q = 0.05 (false discovery rate = 5%; limit 5% of significant results as potential false positives) as Bonferroni multiple comparison correction of p-values was considered too stringent (limit 5% of false positives in the results) [p.adjust(method = ‘BH’)].”

Discussion:

9. The implications of these findings in the context of nicotine dependence is not described. Nor is it placed in the context of what we know about theories around the development and persistence of nicotine seeking.

We expanded the discussion on the parallels between our findings and human fMRI connectivity data that ties to nicotine withdrawal and craving (1, on page 13), the importance of these findings for the theories of nicotine dependence (2, on page 14), and the implication of the findings and the approach used to identify new targets for nicotine dependence and substance use disorder in general (3, on page 16):

(1) “These results are in line with human fMRI data, where increases in resting state connectivity during nicotine withdrawal have also been observed (Fedota and Stein, 2015). Moreover, increased local connectivity within specific network nodes correlate with subjective measures of nicotine craving and measures of nicotine dependence (Claus et al., 2013; Janes et al., 2014; Moran-Santa Maria et al., 2015).”

(2) “The organization of a basal forebrain and a brainstem cholinergic system resembles the original anatomical descriptions of the basal forebrain and brainstem cholinergic systems by Woolf and Mesulam (Mesulam et al., 1983; Woolf, 1991; George et al., 2006), which not only validates the approach of single-cell whole-brain imaging for functional connectome analysis, but has profound implications from a theoretical point of view. Indeed, it suggests that the different cholinergic regions throughout the brain are not independent from each other, but instead are functionally connected through two opposite systems, a basal forebrain cholinergic system and a brainstem cholinergic system. Nicotine withdrawal then emerges with the dysregulation of these two 4 systems that become anticorrelated. Whether one cholinergic system inhibits the other or whether they are anticorrelated though the action of a third system remains to be tested.”

(3) “Finally, these results demonstrate the power of using single-cell whole-brain imaging combined with whole-brain transcriptomics to identify new brain regions, new gene targets, and new cellular pathways that may contribute to nicotine dependence and substance use disorder in general.” 5

Reviewer 2:

This work nicely blends several techniques to comprehensively assess brain changes during nicotine withdrawal. I don’t have major concerns regarding much of the study design, which speaks to the good efforts by the authors. I do have some moderate concerns particularly about how the data is articulated, but these are addressable concerns.

We appreciate both the very positive comments of the reviewer about the muti-domain approach and the reviewer’s concerns regarding the articulation of the data, which helped improve the revised version of our manuscript to be more accessible to a broad audience.

1. In the title please indicate that this work was done in males only. This is relevant given that nicotine has sex specific effects, clinically, behaviorally, and neurobiologically. The fact that this work was done only in males is a major limitation and should be clearly noted. However, it is not a limitation that should bar publication, but it should be noted and discussed as a limitation and indicated in the title so readers do not expect this finding to necessarily translate to females. As suggested “in male mice” was added to the title. This question further overlaps with question 6 in the methods of

Reviewer 1, and was thus addressed by discussing the limitation in the discussion by adding (on page 15): “Finally, another limitation is that both the original whole-brain reactivity (Kimbrough et al., 2021) and whole-brain gene expression (Lein et al., 2007) studies only incorporated male subjects in their study and database precluding any analysis of sex differences. Follow up studies are needed to evaluate if these effects also exist in females.”

2. Can some of the acronyms be regularly spelled out? So many are used that it makes it difficult to hold all that information in mind while reading. Removed the following acronyms by spelling them out in full throughout the manuscript for easier reading (except in some cases when needed in figures/tables; then full names were added to the legend): Ch, FDR, ISH, PC, BFCS, and BSC. Also, except for the main cholinergic regions (MS, PPN, MH, LH, MA, DBN and SI) which are very often used throughout the paper, acronyms for all other brain regions were removed and the regions spelled out completely in the text: ACA, ILA, PL, DP, CP, ACB, BST, BLA, CEA, VTA, IPN, FS, CUN, PS (abbreviations were kept in the figures and full names were added to the figure legend). For improved easy reference throughout the paper, the full names of the 7 main cholinergic regions were added in figure 1. Gene/protein abbreviations were kept, but were spelled out in full on first use.

3. Graph theory jargon makes the impact of the findings difficult to interpret at times. For instance one sentence says “Because the networks are modular, regions that acted as connectors between modules and thus had high participation coefficient (PC) were also considered as hubs?” Can sentences such as these be unpacked a bit more. I think it is important to do this given this work spans different domains of expertise, which is fantastic! However, experts in the molecular neurobiological domain may be less familiar with graph theory terms. The use of jargon to explain other jargon is a barrier and the biological significance of such graph theory metrics may need to be re-articulated in the results. This concern of reviewer 2 aligns with some of the concerns of reviewer 1 in points 1-5, so several clarifications were made already as explained throughout the answers above, in order to explain modularity, hub regions, subcortical-driven network, hyperconnectivity, coordinated activity, and other graph theory related hypotheses. Furthermore, specifically in response to this question: - the within-module degree z-score was clarified (on page 8) as: “which measures the intramodule connectivity or relative importance of a region within its own module” - the specific sentence pointed out on the participation coefficient was rephrased as follows (on page 10): “Because the networks are modular, an important role of hub regions is to act as connectors between modules, which is captured through a high participation coefficient that measures the intermodule connectivity or the extent to which a region connects to multiple other modules. Regions with a high participation coefficient were therefore also considered as hubs.” 6

4. Will information on ∼1700 genes be made available for others to explore further? Yes, with this paper we fully intent to have everything publicly available: FOS counts and gene list will be in tables in the supporting data, as they are currently. The code will be on Github and procedures on lab websites [URLs redacted for double-blind review]. Actually, since the submission additional resources have become available and were added to the manuscript in the Code accessibility section (on page 7), but are currently redacted for review.
