# Supplementary figures and images for: Hyperconnectivity of Two Separate Long-Range Cholinergic Systems Contributes to the Reorganization of the Brain Functional Connectivity during Nicotine Withdrawal in Male Mice
Source: eNeuro. 2023 Jun 26;10(6):ENEURO.0019-23.2023. doi: 10.1523/ENEURO.0019-23.2023 (PMC10306126; doi:10.1523/ENEURO.0019-23.2023)

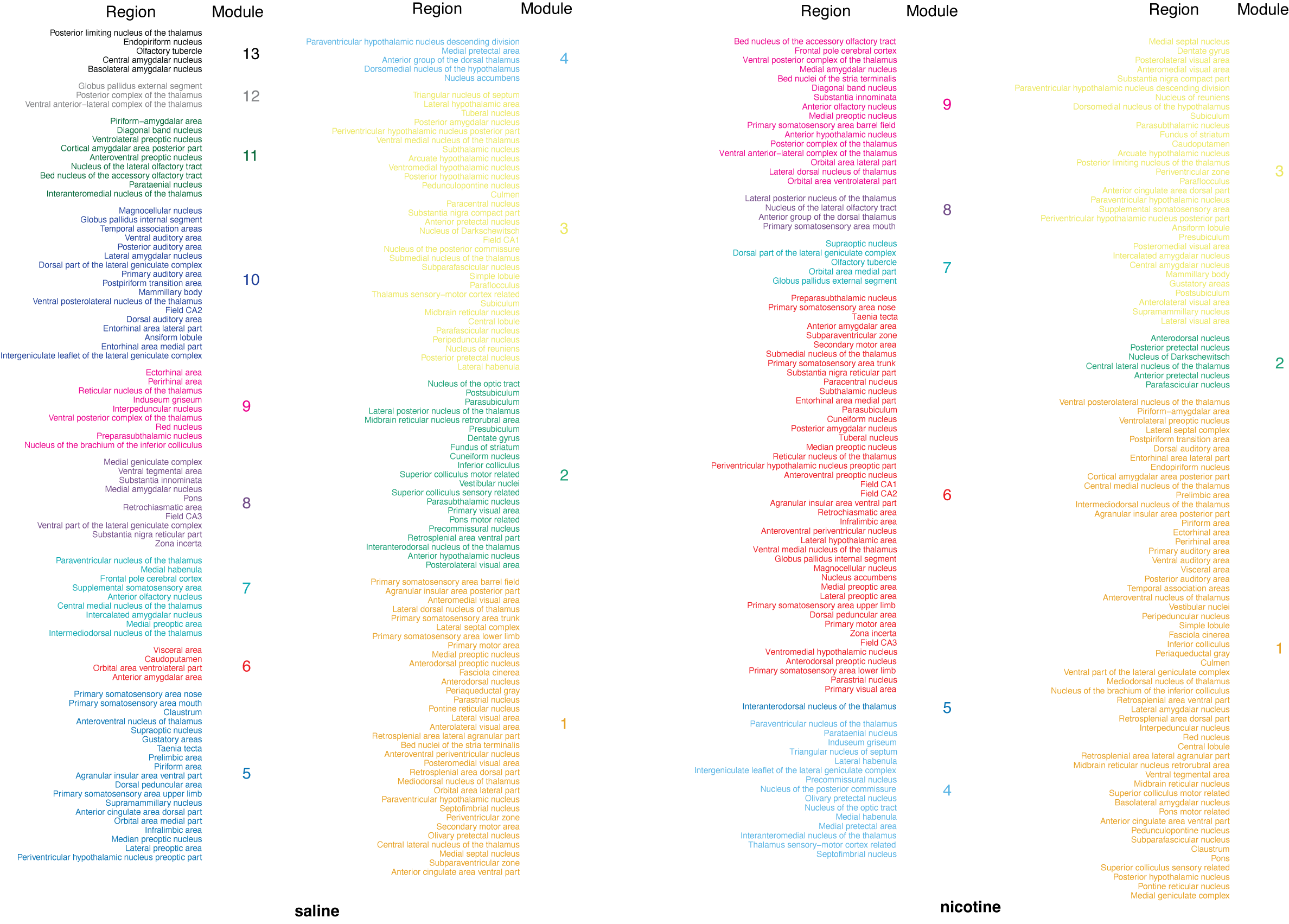

Supplement: Figure 1-1 — Organization of the regions in the clustered correlation matrices for the saline and nicotine groups (Fig. 1B,C) Download Figure 1-1, DOCX file. [file enu-eN-NWR-0019-23-s03.docx]

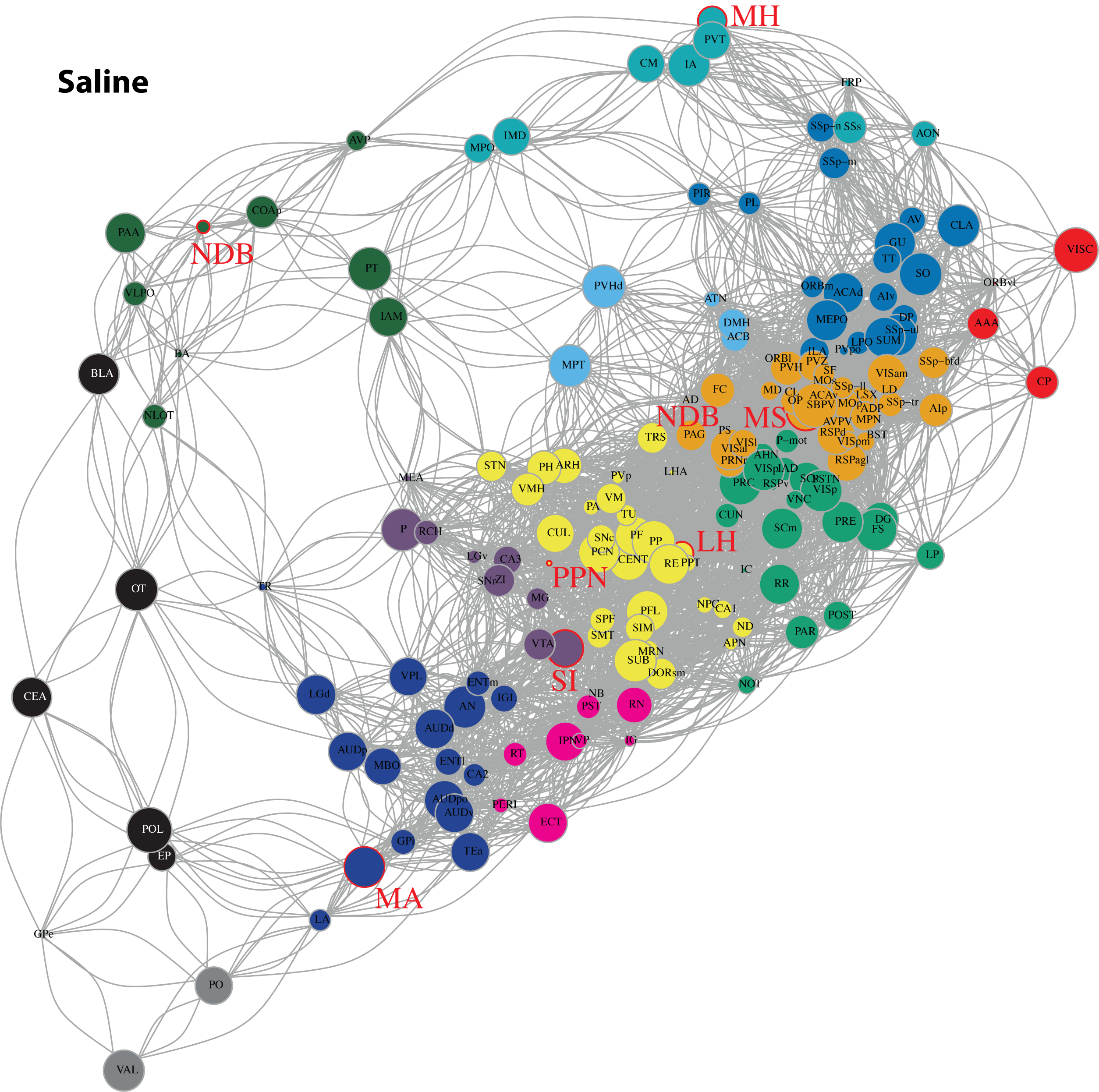

Supplement: Figure 1-2 — Detailed view of Figure 1D with node labels. Download Figure 1-2, DOCX file. [file enu-eN-NWR-0019-23-s04.docx]

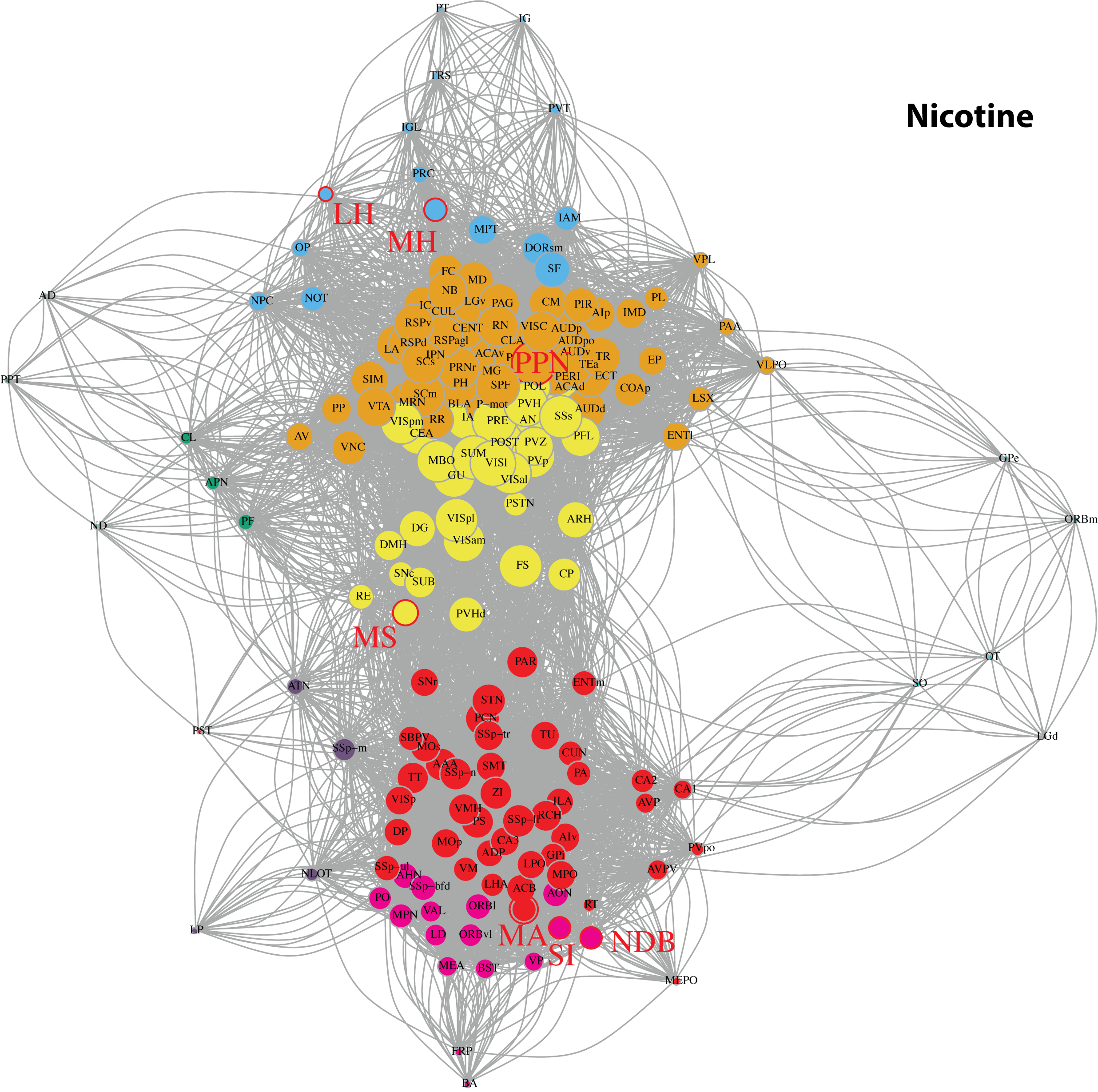

Supplement: Figure 1-3 — Detailed view of Figure 1E with node labels. Download Figure 1-3, DOCX file. [file enu-eN-NWR-0019-23-s05.docx]
