# Supplementary material for: Hyperconnectivity of Two Separate Long-Range Cholinergic Systems Contributes to the Reorganization of the Brain Functional Connectivity during Nicotine Withdrawal in Male Mice
Source: eNeuro. 2023 Jun 26;10(6):ENEURO.0019-23.2023. doi: 10.1523/ENEURO.0019-23.2023 (PMC10306126; doi:10.1523/ENEURO.0019-23.2023)
Supplement: Table 1-1 — Published Fos counts during nicotine withdrawal and saline control. Download Table 1-1, DOCX file. [file enu-eN-NWR-0019-23-s07.docx]

Table 1-1: Published Fos counts during nicotine withdrawal and saline controls

| Region | Abb | Sal1 | Sal2 | Sal3 | Sal4 | Nic1 | Nic2 | Nic3 | Nic4 | Nic5 |
| --- | --- | --- | --- | --- | --- | --- | --- | --- | --- | --- |
| Agranular insular area posterior part | AIp | 496 | 1083 | 1364 | 991 | 1195 | 278 | 549 | 1191 | 991 |
| Agranular insular area ventral part | AIv | 155 | 502 | 408 | 221 | 284 | 494 | 306 | 738 | 550 |
| Ansiform lobule | AN | 47 | 16 | 17 | 75 | 20 | 0 | 17 | 381 | 95 |
| Anterior amygdalar area | AAA | 66 | 88 | 102 | 79 | 92 | 154 | 154 | 286 | 181 |
| Anterior cingulate area dorsal part | ACAd | 294 | 2954 | 1008 | 506 | 798 | 176 | 553 | 2649 | 2315 |
| Anterior cingulate area ventral part | ACAv | 210 | 1947 | 816 | 1159 | 1040 | 95 | 1177 | 3819 | 4540 |
| Anterior group of the dorsal thalamus | ATN | 12 | 23 | 11 | 14 | 3 | 13 | 66 | 69 | 31 |
| Anterior hypothalamic nucleus | AHN | 100 | 407 | 187 | 445 | 53 | 1231 | 500 | 3262 | 544 |
| Anterior olfactory nucleus | AON | 96 | 308 | 587 | 47 | 212 | 892 | 245 | 1427 | 546 |
| Anterior pretectal nucleus | APN | 54 | 83 | 131 | 361 | 44 | 39 | 582 | 371 | 143 |
| Anterodorsal nucleus | AD | 19 | 126 | 17 | 64 | 13 | 4 | 213 | 25 | 25 |
| Anterodorsal preoptic nucleus | ADP | 1 | 12 | 7 | 7 | 8 | 17 | 13 | 24 | 23 |
| Anterolateral visual area | VISal | 38 | 215 | 57 | 210 | 94 | 33 | 99 | 479 | 157 |
| Anteromedial visual area | VISam | 51 | 1388 | 795 | 445 | 290 | 206 | 776 | 4010 | 1444 |
| Anteroventral nucleus of thalamus | AV | 36 | 64 | 60 | 37 | 36 | 23 | 160 | 146 | 91 |
| Anteroventral periventricular nucleus | AVPV | 0 | 46 | 12 | 22 | 20 | 120 | 9 | 284 | 113 |
| Anteroventral preoptic nucleus | AVP | 51 | 22 | 16 | 3 | 18 | 51 | 14 | 153 | 148 |
| Arcuate hypothalamic nucleus | ARH | 44 | 137 | 9 | 131 | 44 | 17 | 27 | 668 | 391 |
| Basolateral amygdalar nucleus | BLA | 700 | 354 | 458 | 266 | 393 | 130 | 403 | 771 | 481 |
| Bed nuclei of the stria terminalis | BST | 125 | 374 | 325 | 344 | 154 | 3701 | 449 | 3124 | 837 |
| Bed nucleus of the accessory olfactory tract | BA | 40 | 13 | 6 | 10 | 7 | 96 | 9 | 42 | 9 |
| Caudoputamen | CP | 5517 | 8131 | 12885 | 7919 | 10055 | 9691 | 10145 | 15966 | 15386 |
| Central amygdalar nucleus | CEA | 565 | 301 | 485 | 291 | 369 | 261 | 475 | 650 | 582 |
| Central lateral nucleus of the thalamus | CL | 7 | 19 | 11 | 14 | 8 | 5 | 217 | 78 | 44 |
| Central lobule | CENT | 74 | 129 | 52 | 340 | 115 | 0 | 111 | 494 | 230 |
| Central medial nucleus of the thalamus | CM | 21 | 29 | 19 | 9 | 16 | 0 | 5 | 22 | 86 |
| Claustrum | CLA | 255 | 568 | 654 | 271 | 454 | 142 | 402 | 683 | 618 |
| Cortical amygdalar area posterior part | COAp | 245 | 159 | 54 | 49 | 244 | 45 | 83 | 501 | 195 |
| Culmen | CUL | 78 | 180 | 16 | 518 | 95 | 0 | 116 | 176 | 290 |
| Cuneiform nucleus | CUN | 2 | 13 | 5 | 27 | 3 | 9 | 6 | 31 | 32 |
| Dentate gyrus | DG | 1305 | 2285 | 3007 | 3272 | 1420 | 1275 | 3347 | 6816 | 3709 |
| Diagonal band nucleus | NDB | 273 | 100 | 41 | 29 | 138 | 658 | 117 | 734 | 475 |
| Dorsal auditory area | AUDd | 2095 | 1282 | 1423 | 2322 | 2239 | 534 | 1004 | 5255 | 3344 |
| Dorsal part of the lateral geniculate complex | LGd | 116 | 9 | 15 | 62 | 89 | 78 | 12 | 146 | 76 |
| Dorsal peduncular area | DP | 16 | 169 | 123 | 42 | 19 | 115 | 89 | 388 | 79 |
| Dorsal premammillary nucleus | PMd | 34 | 10 | 1 | 7 | 0 | 0 | 2 | 149 | 9 |
| Dorsomedial nucleus of the hypothalamus | DMH | 116 | 454 | 118 | 184 | 33 | 31 | 285 | 859 | 213 |
| Ectorhinal area | ECT | 907 | 532 | 1188 | 1734 | 2011 | 262 | 796 | 3920 | 2407 |
| Endopiriform nucleus | EP | 811 | 657 | 793 | 735 | 1240 | 411 | 541 | 1623 | 1150 |
| Entorhinal area lateral part | ENTl | 1009 | 329 | 449 | 1367 | 1110 | 172 | 206 | 2762 | 1061 |
| Entorhinal area medial part | ENTm | 37 | 11 | 9 | 88 | 29 | 34 | 19 | 308 | 84 |
| Fasciola cinerea | FC | 5 | 83 | 6 | 28 | 15 | 0 | 24 | 23 | 93 |
| Field CA1 | CA1 | 1505 | 1937 | 1992 | 3435 | 2148 | 3146 | 1295 | 8391 | 3982 |
| Field CA2 | CA2 | 323 | 218 | 257 | 363 | 310 | 421 | 254 | 780 | 508 |
| Field CA3 | CA3 | 750 | 733 | 587 | 842 | 792 | 1703 | 1111 | 3420 | 1557 |
| Frontal pole cerebral cortex | FRP | 6 | 13 | 13 | 2 | 4 | 1081 | 86 | 151 | 457 |
| Fundus of striatum | FS | 16 | 45 | 73 | 84 | 40 | 33 | 51 | 211 | 115 |
| Globus pallidus external segment | GPe | 139 | 56 | 282 | 72 | 259 | 104 | 42 | 353 | 144 |
| Globus pallidus internal segment | GPi | 28 | 6 | 25 | 36 | 29 | 101 | 31 | 330 | 66 |
| Gustatory areas | GU | 426 | 1312 | 1030 | 471 | 651 | 501 | 858 | 1345 | 978 |
| Induseum griseum | IG | 6 | 3 | 11 | 43 | 47 | 0 | 11 | 1 | 55 |
| Inferior colliculus | IC | 119 | 502 | 398 | 2183 | 234 | 3 | 482 | 663 | 1742 |
| Infralimbic area | ILA | 121 | 1211 | 362 | 385 | 571 | 812 | 545 | 1451 | 965 |
| Interanterodorsal nucleus of the thalamus | IAD | 6 | 21 | 12 | 25 | 7 | 3 | 38 | 0 | 15 |
| Interanteromedial nucleus of the thalamus | IAM | 18 | 20 | 2 | 7 | 9 | 0 | 3 | 4 | 24 |
| Intercalated amygdalar nucleus | IA | 66 | 89 | 59 | 41 | 51 | 25 | 73 | 130 | 109 |
| Intergeniculate leaflet of the lateral geniculate complex | IGL | 41 | 23 | 22 | 64 | 22 | 0 | 33 | 4 | 88 |
| Intermediodorsal nucleus of the thalamus | IMD | 9 | 12 | 6 | 5 | 9 | 0 | 1 | 12 | 33 |
| Interpeduncular nucleus | IPN | 67 | 35 | 76 | 265 | 42 | 0 | 71 | 233 | 50 |
| Lateral amygdalar nucleus | LA | 594 | 328 | 702 | 649 | 747 | 213 | 946 | 1156 | 944 |
| Lateral dorsal nucleus of thalamus | LD | 59 | 97 | 91 | 85 | 48 | 537 | 160 | 830 | 177 |
| Lateral habenula | LH | 46 | 105 | 51 | 256 | 42 | 0 | 247 | 19 | 79 |
| Lateral hypothalamic area | LHA | 608 | 1240 | 556 | 1371 | 977 | 2807 | 923 | 6692 | 1552 |
| Lateral posterior nucleus of the thalamus | LP | 59 | 101 | 202 | 171 | 47 | 359 | 630 | 422 | 187 |
| Lateral preoptic area | LPO | 62 | 312 | 172 | 140 | 117 | 771 | 221 | 1717 | 745 |
| Lateral septal complex | LSX | 260 | 798 | 623 | 628 | 1303 | 584 | 607 | 1796 | 2138 |
| Lateral visual area | VISl | 64 | 233 | 89 | 222 | 33 | 10 | 50 | 307 | 110 |
| Magnocellular nucleus | MA | 22 | 16 | 22 | 23 | 1 | 78 | 6 | 112 | 109 |
| Mammillary body | MBO | 35 | 9 | 12 | 36 | 9 | 0 | 43 | 860 | 53 |
| Medial amygdalar nucleus | MEA | 591 | 409 | 144 | 518 | 167 | 947 | 328 | 873 | 388 |
| Medial geniculate complex | MG | 155 | 126 | 68 | 337 | 85 | 1 | 80 | 584 | 174 |
| Medial habenula | MH | 277 | 410 | 339 | 75 | 117 | 0 | 143 | 31 | 536 |
| Medial preoptic area | MPO | 296 | 359 | 202 | 180 | 156 | 1028 | 276 | 1969 | 981 |
| Medial preoptic nucleus | MPN | 1 | 157 | 49 | 49 | 11 | 349 | 97 | 470 | 181 |
| Medial pretectal area | MPT | 48 | 82 | 29 | 54 | 17 | 0 | 13 | 9 | 69 |
| Medial septal nucleus | MS | 7 | 69 | 25 | 42 | 36 | 50 | 108 | 166 | 166 |
| Median preoptic nucleus | MEPO | 4 | 136 | 26 | 19 | 16 | 66 | 24 | 62 | 193 |
| Mediodorsal nucleus of thalamus | MD | 60 | 189 | 80 | 127 | 161 | 6 | 149 | 173 | 313 |
| Midbrain reticular nucleus | MRN | 324 | 350 | 345 | 864 | 227 | 33 | 510 | 1200 | 561 |
| Midbrain reticular nucleus retrorubral area | RR | 17 | 50 | 77 | 172 | 43 | 4 | 46 | 168 | 34 |
| Nucleus accumbens | ACB | 288 | 954 | 302 | 454 | 214 | 1222 | 445 | 2066 | 1784 |
| Nucleus of Darkschewitsch | ND | 2 | 3 | 4 | 8 | 1 | 0 | 2 | 4 | 0 |
| Nucleus of reunions | RE | 42 | 65 | 42 | 130 | 29 | 32 | 132 | 401 | 68 |
| Nucleus of the brachium of the inferior colliculus | NB | 79 | 64 | 80 | 206 | 78 | 1 | 89 | 105 | 192 |
| Nucleus of the lateral olfactory tract | NLOT | 260 | 30 | 13 | 30 | 3 | 60 | 108 | 107 | 134 |
| Nucleus of the optic tract | NOT | 21 | 32 | 84 | 161 | 21 | 0 | 167 | 35 | 87 |
| Nucleus of the posterior commissure | NPC | 28 | 55 | 50 | 253 | 10 | 0 | 213 | 27 | 41 |
| Olfactory tubercle | OT | 1125 | 49 | 126 | 115 | 1021 | 565 | 142 | 2816 | 474 |
| Olivary pretectal nucleus | OP | 10 | 169 | 38 | 68 | 15 | 0 | 142 | 15 | 68 |
| Orbital area lateral part | ORBl | 22 | 791 | 59 | 139 | 44 | 852 | 167 | 1553 | 306 |
| Orbital area medial part | ORBm | 65 | 719 | 147 | 102 | 314 | 188 | 66 | 313 | 351 |
| Orbital area ventrolateral part | ORBvl | 25 | 371 | 1480 | 109 | 67 | 1068 | 252 | 1371 | 415 |
| Parabigeminal nucleus | PBG | 12 | 10 | 0 | 17 | 9 | 0 | 27 | 11 | 10 |
| Paracentral nucleus | PCN | 5 | 9 | 2 | 23 | 6 | 12 | 9 | 124 | 18 |
| Parafascicular nucleus | PF | 6 | 18 | 3 | 52 | 7 | 7 | 73 | 67 | 21 |
| Paraflocculus | PFL | 10 | 14 | 10 | 45 | 13 | 0 | 2 | 155 | 30 |
| Parastrial nucleus | PS | 3 | 20 | 4 | 15 | 1 | 21 | 11 | 111 | 29 |
| Parasubiculum | PAR | 10 | 18 | 41 | 69 | 8 | 10 | 8 | 440 | 38 |
| Parasubthalamic nucleus | PSTN | 18 | 41 | 34 | 47 | 17 | 5 | 10 | 70 | 11 |
| Parataenial nucleus | PT | 267 | 267 | 139 | 186 | 182 | 11 | 113 | 16 | 221 |
| Paraventricular hypothalamic nucleus | PVH | 38 | 405 | 85 | 135 | 101 | 21 | 98 | 467 | 399 |
| Paraventricular hypothalamic nucleus descending division | PVHd | 22 | 64 | 12 | 22 | 18 | 19 | 32 | 104 | 38 |
| Paraventricular nucleus of the thalamus | PVT | 600 | 884 | 864 | 95 | 689 | 0 | 31 | 9 | 1019 |
| Pedunculopontine nucleus | PPN | 22 | 23 | 16 | 31 | 11 | 0 | 8 | 47 | 32 |
| Periaqueductal gray | PAG | 172 | 636 | 171 | 503 | 333 | 0 | 195 | 857 | 595 |
| Peripeduncular nucleus | PP | 7 | 12 | 6 | 22 | 1 | 0 | 15 | 15 | 38 |
| Perirhinal area | PERI | 1122 | 691 | 1114 | 1648 | 1295 | 259 | 769 | 2652 | 1793 |
| Periventricular hypothalamic nucleus posterior part | PVp | 12 | 40 | 5 | 49 | 11 | 0 | 6 | 189 | 22 |
| Periventricular hypothalamic nucleus preoptic part | PVpo | 2 | 112 | 26 | 18 | 8 | 38 | 13 | 80 | 203 |
| Periventricular zone | PVZ | 7 | 208 | 41 | 50 | 37 | 19 | 46 | 171 | 226 |
| Piriform area | PIR | 3522 | 4826 | 3418 | 3316 | 5806 | 1158 | 2281 | 5829 | 5116 |
| Piriform-amygdalar area | PAA | 341 | 74 | 32 | 24 | 852 | 12 | 28 | 657 | 173 |
| Pons | P | 369 | 312 | 217 | 358 | 439 | 2 | 219 | 2845 | 1220 |
| Pons motor related | P-mot | 39 | 226 | 92 | 221 | 62 | 0 | 77 | 1433 | 273 |
| Pontine reticular nucleus | PRNr | 51 | 119 | 61 | 124 | 65 | 0 | 76 | 510 | 195 |
| Posterior amygdalar nucleus | PA | 21 | 43 | 9 | 61 | 22 | 36 | 19 | 133 | 64 |
| Posterior auditory area | AUDpo | 88 | 7 | 36 | 150 | 193 | 2 | 47 | 591 | 378 |
| Posterior complex of the thalamus | PO | 20 | 5 | 93 | 30 | 3 | 245 | 66 | 773 | 40 |
| Posterior hypothalamic nucleus | PH | 303 | 441 | 164 | 450 | 77 | 0 | 123 | 1122 | 384 |
| Posterior limiting nucleus of the thalamus | POL | 12 | 7 | 11 | 9 | 7 | 3 | 8 | 14 | 24 |
| Posterior pretectal nucleus | PPT | 3 | 10 | 4 | 38 | 3 | 0 | 89 | 14 | 2 |
| Posterolateral visual area | VISpl | 6 | 37 | 14 | 45 | 1 | 0 | 9 | 117 | 20 |
| posteromedial visual area | VISpm | 9 | 1431 | 442 | 760 | 156 | 22 | 852 | 3071 | 1680 |
| Postpiriform transition area | TR | 223 | 28 | 18 | 69 | 89 | 8 | 22 | 206 | 168 |
| Postsubiculum | POST | 33 | 94 | 342 | 584 | 139 | 59 | 154 | 494 | 308 |
| Precommissural nucleus | PRC | 29 | 72 | 41 | 85 | 26 | 0 | 23 | 6 | 44 |
| Prelimbic area | PL | 279 | 1451 | 368 | 211 | 559 | 250 | 308 | 595 | 1010 |
| Preparasubthalamic nucleus | PST | 6 | 5 | 6 | 12 | 3 | 3 | 3 | 22 | 1 |
| Presubiculum | PRE | 61 | 282 | 371 | 588 | 152 | 23 | 160 | 1080 | 423 |
| Primary auditory area | AUDp | 1664 | 647 | 819 | 1486 | 2230 | 202 | 771 | 3565 | 2952 |
| Primary motor area | MOp | 716 | 14331 | 5991 | 6321 | 2814 | 11439 | 7902 | 23397 | 11076 |
| Primary somatosensory area barrel field | SSp-bfd | 1286 | 3242 | 3670 | 2285 | 1966 | 7040 | 4277 | 10541 | 4580 |
| Primary somatosensory area lower limb | SSp-ll | 295 | 5333 | 2162 | 2104 | 1110 | 2750 | 1775 | 4944 | 3859 |
| Primary somatosensory area mouth | SSp-m | 1073 | 4813 | 2147 | 711 | 850 | 1896 | 2933 | 4947 | 2214 |
| Primary somatosensory area nose | SSp-n | 1465 | 3286 | 2158 | 948 | 1341 | 2354 | 2290 | 5771 | 2622 |
| Primary somatosensory area trunk | SSp-tr | 56 | 2415 | 1502 | 958 | 477 | 1125 | 1283 | 3297 | 2578 |
| Primary somatosensory area upper limb | SSp-ul | 688 | 5664 | 3453 | 1863 | 927 | 4609 | 3311 | 7481 | 4302 |
| Primary visual area | VISp | 103 | 1793 | 918 | 3118 | 196 | 1672 | 1656 | 7180 | 2810 |
| Red nucleus | RN | 24 | 19 | 30 | 109 | 48 | 0 | 35 | 151 | 60 |
| Reticular nucleus of the thalamus | RT | 90 | 22 | 65 | 401 | 45 | 150 | 126 | 198 | 381 |
| Retrochiasmatic area | RCH | 190 | 81 | 10 | 176 | 52 | 250 | 89 | 1099 | 550 |
| Retrosplenial area dorsal part | RSPd | 107 | 3384 | 1282 | 1977 | 505 | 0 | 2341 | 5927 | 2977 |
| Retrosplenial area lateral agranular part | RSPagl | 47 | 1668 | 829 | 2169 | 547 | 0 | 954 | 5342 | 1827 |
| Retrosplenial area ventral part | RSPv | 228 | 2604 | 901 | 4150 | 1273 | 6 | 2906 | 5708 | 3226 |
| Secondary motor area | MOs | 645 | 7208 | 2528 | 2831 | 1740 | 4008 | 5508 | 10852 | 6794 |
| Septofimbrial nucleus | SF | 13 | 186 | 54 | 75 | 97 | 19 | 53 | 78 | 139 |
| Simple lobule | SIM | 20 | 40 | 22 | 728 | 15 | 1 | 156 | 218 | 239 |
| Subiculum | SUB | 252 | 270 | 279 | 1280 | 244 | 277 | 849 | 1986 | 811 |
| Submedial nucleus of the thalamus | SMT | 7 | 7 | 5 | 28 | 2 | 11 | 10 | 41 | 33 |
| Subparafascicular nucleus | SPF | 73 | 81 | 62 | 180 | 59 | 6 | 60 | 269 | 197 |
| Subparaventricular zone | SBPV | 5 | 248 | 56 | 90 | 7 | 40 | 76 | 222 | 156 |
| Substantia innominata | SI | 177 | 170 | 163 | 208 | 176 | 941 | 148 | 1496 | 397 |
| Substantia nigra compact part | SNc | 21 | 34 | 11 | 66 | 12 | 8 | 31 | 246 | 20 |
| Substantia nigra reticular part | SNr | 86 | 59 | 26 | 117 | 22 | 45 | 106 | 1022 | 97 |
| Subthalamic nucleus | STN | 30 | 36 | 14 | 36 | 9 | 18 | 20 | 152 | 42 |
| Superior colliculus motor related | SCm | 253 | 1169 | 890 | 2795 | 414 | 44 | 995 | 2742 | 1486 |
| Superior colliculus sensory related | SCs | 48 | 1520 | 602 | 2794 | 244 | 3 | 387 | 1633 | 706 |
| Supplemental somatosensory area | SSs | 3479 | 5752 | 5353 | 2603 | 3489 | 1380 | 2529 | 6370 | 3864 |
| Suprachiasmatic nucleus | SCH | 0 | 184 | 14 | 23 | 81 | 80 | 79 | 157 | 493 |
| Supramammillary nucleus | SUM | 8 | 32 | 22 | 16 | 6 | 0 | 12 | 218 | 28 |
| Supraoptic nucleus | SO | 2 | 33 | 30 | 4 | 40 | 44 | 4 | 97 | 248 |
| Taenia tecta | TT | 151 | 755 | 474 | 177 | 194 | 478 | 513 | 1159 | 601 |
| Temporal association areas | TEa | 1914 | 788 | 2007 | 2818 | 3272 | 336 | 1457 | 6394 | 4439 |
| Thalamus sensory-motor cortex related | DORsm | 4 | 5 | 6 | 45 | 15 | 0 | 5 | 9 | 40 |
| Triangular nucleus of septum | TRS | 14 | 207 | 5 | 143 | 48 | 0 | 28 | 2 | 93 |
| Tuberal nucleus | TU | 78 | 167 | 50 | 257 | 94 | 171 | 94 | 1184 | 372 |
| Ventral anterior-lateral complex of the thalamus | VAL | 25 | 21 | 42 | 30 | 19 | 95 | 44 | 185 | 39 |
| Ventral auditory area | AUDv | 1953 | 1330 | 1815 | 2191 | 2663 | 381 | 1079 | 4339 | 3884 |
| Ventral medial nucleus of the thalamus | VM | 79 | 127 | 55 | 152 | 86 | 320 | 92 | 1094 | 166 |
| Ventral part of the lateral geniculate complex | LGv | 219 | 194 | 132 | 239 | 174 | 3 | 137 | 210 | 289 |
| Ventral posterior complex of the thalamus | VP | 38 | 24 | 39 | 102 | 36 | 435 | 44 | 634 | 81 |
| Ventral posterolateral nucleus of the thalamus | VPL | 104 | 31 | 30 | 126 | 64 | 17 | 21 | 44 | 48 |
| Ventral tegmental area | VTA | 199 | 135 | 94 | 495 | 83 | 1 | 438 | 1789 | 168 |
| Ventrolateral preoptic nucleus | VLPO | 67 | 8 | 4 | 1 | 76 | 22 | 18 | 84 | 93 |
| Ventromedial hypothalamic nucleus | VMH | 40 | 71 | 13 | 75 | 30 | 194 | 93 | 1267 | 159 |
| Vestibular nuclei | VNC | 20 | 90 | 57 | 137 | 7 | 0 | 152 | 428 | 32 |
| Visceral area | VISC | 866 | 1114 | 1762 | 974 | 1073 | 248 | 655 | 1578 | 1124 |
| Zona incerta | ZI | 348 | 277 | 151 | 464 | 227 | 520 | 360 | 1296 | 559 |
