# Supplementary material for: Hyperconnectivity of Two Separate Long-Range Cholinergic Systems Contributes to the Reorganization of the Brain Functional Connectivity during Nicotine Withdrawal in Male Mice
Source: eNeuro. 2023 Jun 26;10(6):ENEURO.0019-23.2023. doi: 10.1523/ENEURO.0019-23.2023 (PMC10306126; doi:10.1523/ENEURO.0019-23.2023)
Supplement: Supplementary file 4 [file enu-eN-NWR-0019-23-s06.docx]

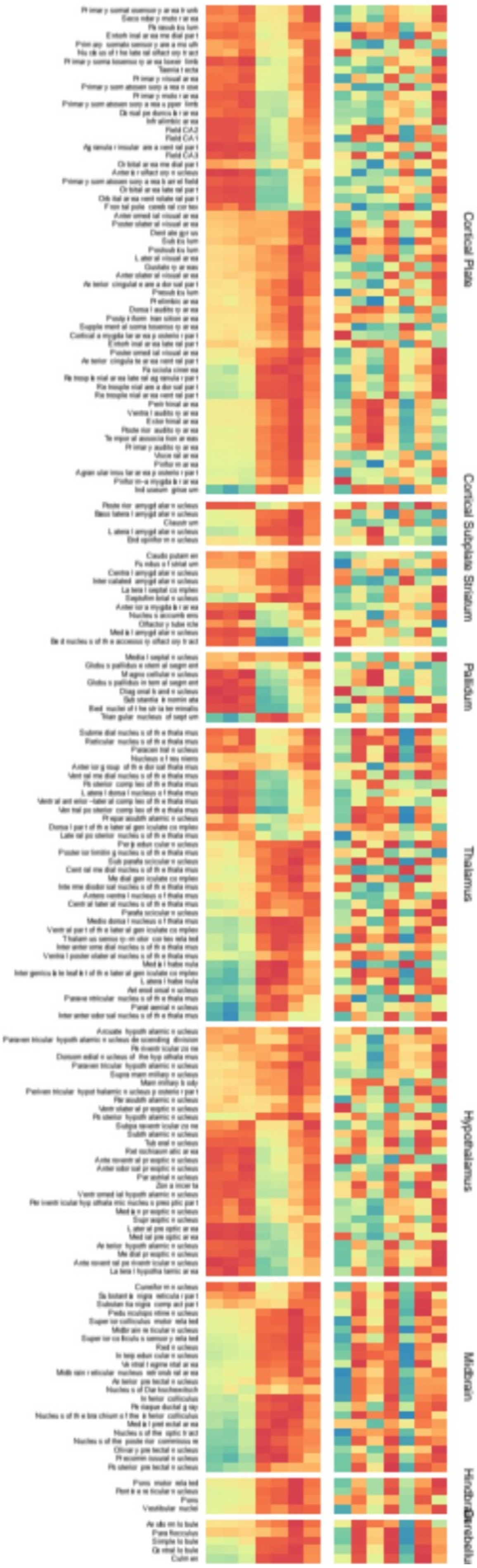
Cortical Plate
Cortical Subplate
Striatum
Pallidum
Thalamus
Hypothalamus
Midbrain
